# Noncanonical lipooligosaccharide assembly in *Acinetobacter baumannii* is mediated by the glycosyltransferases KdoT and GnaT

**DOI:** 10.1016/j.jbc.2025.111103

**Published:** 2025-12-23

**Authors:** Leah M. VanOtterloo, Bradley J. Voss, M. Stephen Trent

**Affiliations:** 1 Department of Microbiology, College of Art and Sciences, University of Georgia, Athens, Georgia, USA; 2 Department of Infectious Diseases, College of Veterinary Medicine, University of Georgia, Athens, Georgia, USA

**Keywords:** *Acinetobacter baumannii*, lipooligosaccharide, LOS, core-oligosaccharide, Kdo, GlcNAcA, lipid A

## Abstract

The asymmetric outer membrane is a defining feature of Gram-negative bacteria that provides essential barrier function. The inner leaflet contains glycerophospholipids whereas the outer leaflet is composed of lipopolysaccharide or lipooligosaccharide (LOS). Lipopolysaccharide is comprised of a lipid A anchor, core oligosaccharide (core OS), and O-antigen, while LOS lacks the O-antigen component. Modifications to any of these elements alter barrier permeability. *Acinetobacter baumannii* demonstrates an unusual ability to survive in the absence of LOS, which offers resistance against select antibiotics but forfeits the outer membrane integrity afforded by LOS. Despite this important relationship, the steps involved in building the core OS component of *A. baumannii* LOS remain incompletely described. Here, we complete the elucidation of this pathway by establishing a unique method of KdoIII addition *via* the glycosyltransferase KdoT followed by GlcNAcA addition *via* GnaT—a clear departure from the typical WaaA-only model of consecutive Kdo transfer. We reconstituted *in vitro* a two-step sequence in which KdoT transfers the final Kdo residue (KdoIII) and GnaT subsequently transfers GlcNAcA. Heterologous expression confirmed the presence of KdoT homologs across several Gram-negative species, indicating that this split Kdo pathway is not unique to *A. baumannii*. Structural modeling and targeted mutagenesis further examined the glycosyltransferase assignments of KdoT and GnaT and probed the potential mechanisms employed by each. Together, these data complete the early core OS synthesis pathway in *A. baumannii* by establishing a noncanonical two-enzyme mechanism for inner core Kdo transfer followed by GlcNAcA addition.

The hallmarked outer membrane of Gram-negative bacteria is a uniquely asymmetric and selectively permeable barrier that provides intrinsic resistance to many antibiotics (1, 2, 3). While the inner leaflet of the outer membrane is mostly composed of glycerophospholipids, the outer leaflet is fortified by lipopolysaccharide (LPS), a negatively charged molecule composed of a lipid A anchor, core oligosaccharide (core OS), and O-antigen (4, 5). Many organisms often lack the O-antigen component, resulting in lipooligosaccharide (LOS). Whether present as LPS or LOS, barrier function of the bacterial outer membrane is conferred by common structural features of the molecule. While densely packed hydrophobic acyl chains found in the lipid A moiety help form a strong membrane foundation, negative charges contributed by lipid A phosphates and phosphorylated or acidic sugar residues found in the core OS create a highly anionic surface that coordinates divalent cations to form an intricate and compact lattice (6, 7). These interactions across the outer leaflet reinforce the outer membrane and severely limit the penetration of hydrophobic and amphipathic toxins (8). As such, structural modifications to any of these components directly reshape lateral interactions and influences bacterial fitness.

A noteworthy example of this relationship between LPS/LOS modifications and bacterial fitness can be seen in *Acinetobacter baumannii*, a Gram-negative bacterium whose rapid evolution of antibiotic defense strategies has led to its classification as a high-priority pathogen (9) (https://www.cdc.gov/antimicrobial-resistance/data-research/threats/index.html). In addition to well-studied lipid A modifications that alter the charge of its LOS and the cell surface at large, *A. baumannii* has a unique ability to completely disrupt LOS synthesis as a resistance mechanism against LOS-targeting cationic antimicrobial peptides (10, 11). Although this drastic remodeling confers drug resistance, it directly compromises outer membrane integrity and imposes severe fitness costs (12). This clear cause-and-effect relationship makes *A. baumannii* a powerful tool in studying how LPS/LOS governs the Gram-negative outer membrane.

Given the importance of the molecule, it is essential to understand the mechanisms that assemble LOS. *A. baumannii* LOS assembly, like all Gram-negative organisms, is conducted on the cytoplasmic face of the inner membrane (13). A multi-enzyme process known as the Raetz pathway for lipid A synthesis begins with UDP-N-acetyl-glucosamine (UDP-GlcNAc). Synthesis continues *via* a series of enzymes that convert this molecule to lipid IV_A_, after which the Kdo transferase WaaA (KdtA) attaches 3-deoxy-D-*manno*-octulosonic acid (Kdo) residues to yield Kdo_2_-lipid IV_A_ (Fig. 1*A*). These Kdo sugars represent the first steps of core OS synthesis. Late lipid A synthesis steps result in the addition of secondary lipid A acyl chains, yielding a complete Kdo_2_–lipid A molecule.

**Figure 1:**
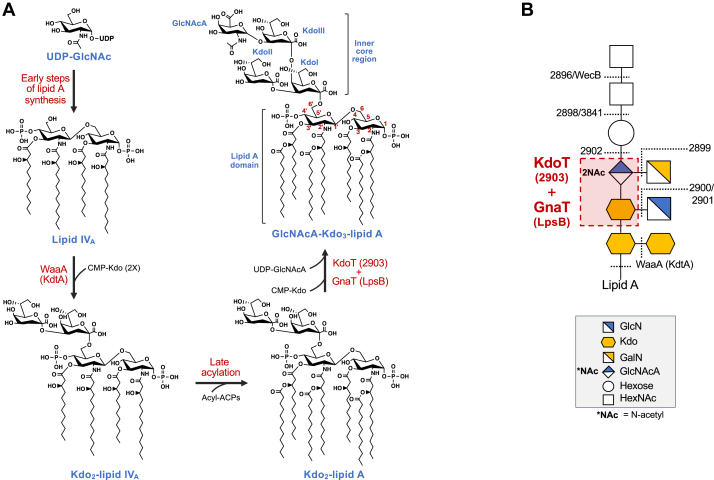
**Early core oligosaccharide assembly in *Acinetobacter baumannii*.***A*, pathway overview of early lipid A and core OS synthesis. Early lipid A synthesis from UDP-GlcNAc yields a *bis*-phosphorylated lipid IV_A_ precursor. The Kdo transferase WaaA (also known as KdtA) begins core OS synthesis *via* the transfer of Kdo residues using CMP-Kdo as the donor to form Kdo_2_-lipid IV_A_. Late acylation steps complete Kdo_2_-lipid A assembly using acyl-ACPs as donor substrates. The addition of the acyloxyacyl-linked fatty acid at the 2-position, however, is partial (*dashed* bond) resulting in both hexa- and hepta-acylated Kdo_2_-lipid A species in *A. baumannii*. Core OS is extended *via* transfer of CMP-Kdo and UDP-GlcNAcA by two proteins, KdoT and GnaT, characterized here (16). The KdoIII residue added by KdoT diverges from inner core assembly in other bacteria, as WaaA is normally responsible for the transfer of all Kdo sugars. The core OS is then extended by a series of enzymes not shown in the schematic, completing LOS assembly. *B*, diagram of the complete LOS structure of *A. baumannii* ATCC 17978. *Dashed lines* indicate proteins predicted to transfer their corresponding residues based on knockout mutation analysis and prior literature (16, 54). Note that deletion of either *kdoT* or *gnaT* results in an identical Kdo_2_–lipid A chemotype and expression of both proteins is required for KdoIII and GlcNAcA addition (*red box*) in whole cells. For most strains characterized to date, the inner core structure of *A. baumannii* LOS is conserved. Sugar residues are depicted using the official Symbol Nomenclature for Glycans (SNFG) (55).

Despite extensive studies surrounding the synthesis and alteration of lipid A, the core OS of *A. baumannii* has been comparatively understudied. Several bioinformatic and genetic studies (14, 15, 16), however, have begun to map the core OS structure and identify important polysaccharide synthesis loci likely involved in core OS synthesis (Fig. 1*B*). Our previous work confirmed the role of such a locus by systematically constructing targeted mutants and defining their LOS chemotypes, allowing us to assign provisional functions to predicted core OS glycosyltransferases and associated enzymes. Among these identified genes, two early core OS genes stand out—*2903* (*A1S_2903*) and *lpsB* (*A1S_0430*). We discovered that both genes are necessary for the production of a fully extended inner core, and loss of either yields identical LOS chemotypes with a core OS containing only two Kdo sugars. Importantly, both KdoIII and N-acetylglucosaminuronic acid (GlcNAcA) additions depend on the presence of both 2903 and LpsB in the cell, implicating these two enzymes in KdoIII and GlcNAcA transfer.

These observations were unusual for multiple reasons. First, core OS glycosyltransferases are typically relegated to single-sugar transfers, making dual dependence for two distinct additions atypical (17). The mechanism by which these two sugars are added and how 2903 and LpsB function in this transfer is not currently known. Second, and central to the field’s understanding of the enzyme, WaaA alone typically transfers all contiguous inner core Kdo residues (18, 19, 20). For example, WaaA of *Escherichia coli* is bifunctional and transfers two Kdo residues to fulfill the Kdo disaccharide consistently found in its core OS (18). In contrast, WaaA of *Haemophilus influenzae* is monofunctional and transfers a single Kdo, while the homolog in *Chlamydia trachomatis* is trifunctional and transfers three Kdo residues (19, 20). While other core OS Kdo transferases have been described, they are either involved in outer core synthesis—not synthesis of the contiguous inner core Kdo bloc—or modification enzymes not typically involved in WT core OS synthesis (21, 22). The requirement for a second enzyme to transfer KdoIII in *A. baumannii* and complete the Kdo trisaccharide therefore represents a departure from the WaaA-only paradigm and suggests a division of labor in inner core Kdo addition not previously described in Gram-negative bacteria.

Here, we resolve the specific roles of 2903 and LpsB in *A. baumannii* core OS assembly. We define their order of action relative to early core OS synthesis, define donor-acceptor specificities, and map homologs in a distant Gram-negative class. We further leverage structural predictions and comparative bioinformatics to contextualize their glycosyltransferase features and putative active site architecture. Together, these data support a model for a previously unrecognized mechanism of core OS Kdo transfer. For clarity of nomenclature, we hereafter refer to A1S_2903 as KdoT, the KdoIII transferase, and to LpsB as GnaT, the GlcNAcA transferase.

## Results

### Purification of KdoT and GnaT

**Figure 2:**
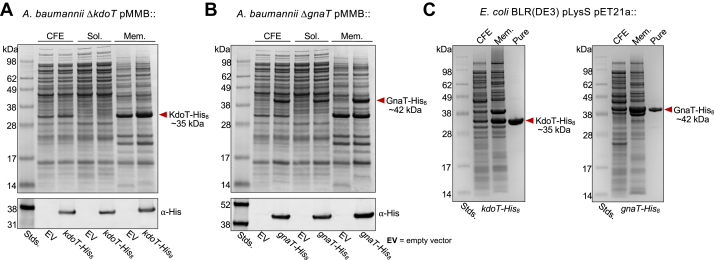
**Cellular localization and purification of KdoT and GnaT proteins.***A*, *Top*: SDS-PAGE with Coomassie staining of cell-free extract (CFE), soluble, and membrane fractions of *Acinetobacter baumannii* Δ*kdoT* with empty vector (EV) or expressing KdoT-His_8_ (∼35 kDa) to identify subcellular localization. Bottom: Western blot of KdoT-His_8_. *B*, *Top*: SDS-PAGE with Coomassie staining of cell-free extract (CFE), soluble, and membrane fractions of *A. baumannii* Δ*gnaT* with empty vector or expressing GnaT-His_8_ (∼42 kDa) to identify subcellular localization. *Bottom*: Western blot of GnaT-His_8_. Data in (*A*) and (*B*) are representative of three biological replicates. *C*, SDS-PAGE with Coomassie staining of cell-free extract (CFE), membrane fractions, and purified proteins isolated from *Escherichia coli* BLR (DE3) pLysS containing either pET21a::*kdoT-His*_*8*_ or::*gnaT*-*His*_*8*_.

The most direct investigation of KdoT and GnaT function required purification of both proteins. To maximize protein yield, we first identified the subcellular localization of each by encoding C-terminally His_8_-tagged KdoT and GnaT into individual pMMB67EH vectors and expressing them in *A. baumannii* 17,978 Δ*kdoT* and Δ*gnaT*, respectively. A small portion of cell-free extract was collected from each culture while the remainder was further separated into soluble and membrane fractions to be analyzed by SDS-PAGE. Both Coomassie staining and anti-histidine immunoblotting revealed the presence of KdoT-His_8_ (∼35 kDa) and GnaT-His_8_ (∼42 kDa) in both the soluble and membrane fractions (Fig. 2, *A* and *B*). Although neither protein has any predicted transmembrane helices according to bioinformatic analysis (23), they are clearly membrane associated, a trait not uncommon among LPS synthesis enzymes (24, 25, 26).

Before moving to purification, we investigated if the His-tag impacted protein function and LOS assembly. To do this, we treated whole cell lysate from mutant strains (Δ*kdoT* or Δ*gnaT*) expressing our target proteins with proteinase K, then separated the lysate by SDS-PAGE and visualized the LOS pattern *via* Pro-Q Emerald 300 carbohydrate staining (Fig. S1). According to these results, the tag did not alter enzymatic function compared to their untagged counterparts. It should be noted that while expression of either GnaT-His_8_ or untagged GnaT restored core OS to the WT pattern, expression of either KdoT-His_8_ or untagged KdoT restored core OS to a majority intermediate length while producing only a small amount of full-length core OS. This pattern was previously reported for KdoT complementation and is likely due to stoichiometric imbalance or differing rates of inner core *versus* outer core synthesis (16).

To purify each protein to near-homogeneity, *kdoT* and *gnaT* were cloned into pET21a vectors with a C-terminal His_8_-tag and overexpressed in *E. coli* BLR(DE3) pLysS. Membrane fractions containing the proteins of interest were solubilized and the proteins purified *via* affinity chromatography (see Experimental Procedures for details). Proteins of expected sizes corresponding to KdoT-His_8_ and GnaT-His_8_ were clearly observed and highly abundant based on SDS-PAGE, proving successful purification of each protein (Fig. 2*C*).

### KdoT is a CMP-Kdo transferase

**Figure 3:**
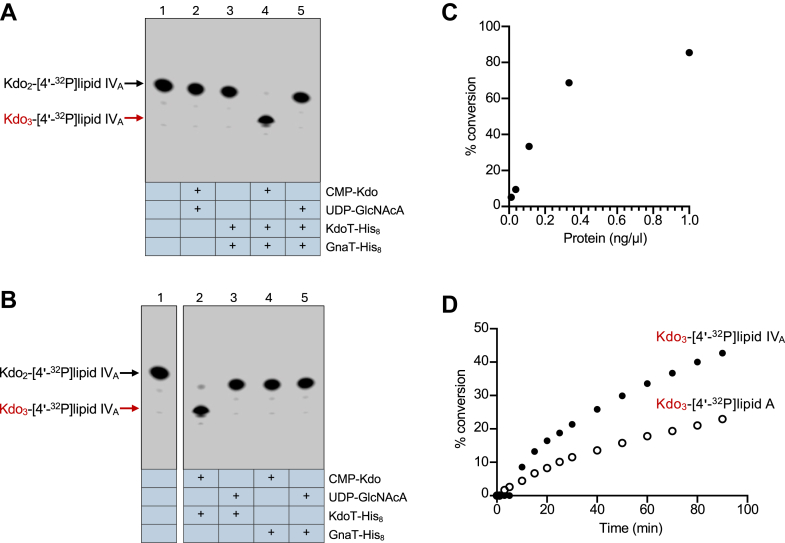
**KdoT is a CMP-Kdo transferase.***A* and *B*, TLC of *in vitro* reactions containing Kdo_2_-[4′-^32^P]lipid IV_A_ (lane 1) plus indicated combinations of KdoT-His_8_, GnaT-His_8_, CMP-Kdo, and UDP-GlcNAcA (lanes 2–5). Kdo_3_-[4′-^32^P]lipid IV_A_ migrates below the substrate (*red arrow*). In (*A*), both enzymes were present while the sugar donors were modulated. Activity was observed in the presence of both enzymes and CMP-Kdo. In (*B*), both the enzymes and sugar donors were modulated. Note that lane 1, lipid-only control, is copied from *panel A*. Activity was observed in the reaction containing only KdoT-His_8_ and CMP-Kdo, confirming KdoT as the CMP-Kdo transferase. No activity was seen when GnaT-His_8_ was used as the sole enzyme. Data in (*A*) and (*B*) are representative of three independent experiments. *C*, conversion of Kdo_2_-[4′-^32^P]lipid IV_A_ to Kdo_3_-[4′-^32^P]lipid IV_A_ as a function of KdoT concentration under standard conditions. Product conversion was quantified *via* TLC densitometry. *D*, conversion of Kdo_2_-[4′-^32^P]lipid IV_A_ to Kdo_3_-[4′-^32^P]lipid IV_A_ (*closed circles*) and hexa-acylated Kdo_2_-[4′-^32^P]lipid A to Kdo_3_-[4′-^32^P]lipid A (*open circles*) over time under standard conditions. Product conversion was quantified *via* TLC densitometry.

It was previously discovered by our group that while both *kdoT* and *gnaT* mutants had identical Kdo_2_–lipid A chemotypes, co-expression of both genes facilitated the addition of KdoIII and GlcNAcA residues to the core OS of an *A. baumannii* mutant synthesizing only Kdo_2_-lipid A (16). Expression of either gene alone showed no effect on chemotype. To investigate this transfer mechanism, yet keeping in mind that both proteins appear to be required for activity, we designed *in vitro* transferase assays using purified KdoT-His_8_ and GnaT-His_8_ as enzyme sources, but with only single nucleotide-activated sugars as donors. This approach allowed us to determine whether both CMP-Kdo and UDP-GlcNAcA residues were required for enzymatic function. Because CMP-Kdo has a relatively short half-life, it was generated *in situ via* purified CMP-Kdo synthetase (KdsB) to ensure adequate substrate availability as previously described (21, 27). UDP-GlcNAcA, conversely, was synthesized in advance and provided exogenously in excess. Kdo_2_-lipid IV_A_ labeled with ^32^P at the 4′-phosphate group (Kdo_2_-[4′-^32^P]lipid IV_A_) was provided as the acceptor substrate (28). Reaction products were separated by thin-layer chromatography (TLC) in a solvent system of chloroform, pyridine, 88% formic acid, water (30:70:16:10, v/v) and analyzed by phosphorimaging. In this system, addition of a sugar slows migration, resulting in a downward shift in the lipid substrate (20, 29). Indeed, phosphorimaging revealed that the reaction containing only CMP-Kdo and both KdoT-His_8_ and GnaT-His_8_ resulted in a product relative to the Kdo_2_-[4′-^32^P]lipid IV_A_–only control, indicative of Kdo addition (Fig. 3*A*, lane 4). No activity was seen when UDP-GlcNAcA was provided as the only sugar donor, which was unsurprising as the KdoIII residue precedes GlcNAcA based on existing structural data (15, 16). These results demonstrate that transfer of KdoIII is not dependent on the presence of GlcNAcA *in vitro* and suggests only one sugar residue is transferred at a time in *A. baumannii* during core OS synthesis.

Despite their apparent reliance on each other in the cell, KdoT and GnaT are not predicted to interact with each other based on *in vivo* immunoprecipitation assays as well as AlphaFold3 protein–protein modeling (Fig. S2). We were therefore curious to see if either protein would be able to act alone. In the reaction containing only CMP-Kdo and KdoT-His_8_, we also observed a product indicative of Kdo sugar transfer (Fig. 3*B*, lane 2) demonstrating that KdoT can function in the absence of GnaT *in vitro*. No product shift was seen using any other single enzyme-sugar combinations (lanes 3–5). Similar outcomes were observed when hexa-acylated Kdo_2_-[4′-^32^P]lipid A was used as the substrate *in lieu* of tetra-acylated Kdo_2_-[4′-^32^P]lipid IV_A_ (Fig. S3*A*).

The enzymatic parameters of KdoT were explored further by assessing its ability to facilitate conversion of Kdo_2_-[4′-^32^P]lipid IV_A_ substrate to Kdo_3_-[4′-^32^P]lipid IV_A_ product in the presence of CMP-Kdo. Measurements were made by TLC densitometry. We first examined the effect of enzyme concentration on product formation, which revealed that conversion of the substrate increased with enzyme concentration until a plateau was approached at enzyme concentrations greater than 0.4 ng/μl (Fig. 3*C*). We then monitored product formation over a course of 90 min using a constant protein concentration. When measured as a function of time, KdoT displays linear activity typical of an enzyme (Fig. 3*D*). This assay also revealed a clear preference of KdoT for Kdo_2_-lipid IV_A_ compared to hexa-acylated Kdo_2_-lipid A, with approximately 40% conversion to Kdo_3_-lipid IV_A_ achieved after 90 min *versus* only 20% conversion to Kdo_3_-lipid A in the same amount of time.

Since WaaA normally transfers Kdo from CMP-Kdo to the lipid IV_A_ precursor to initiate core OS synthesis (Fig. 1*A*), we asked whether KdoT could substitute for this activity and add Kdo directly to lipid IV_A._ We generated a [4′-^32^P]lipid IV_A_ substrate lacking Kdo residues and performed *in vitro* assays and TLC separation as before to compare previously purified bifunctional WaaA activity with various combinations of enzymes and sugar donors including KdoT-His_8_ and CMP-Kdo (30, 31). The reaction containing WaaA and CMP-Kdo resulted in a dramatic downward product shift indicating Kdo addition as expected (Fig. S4). However, no other enzyme-donor combinations resulted in sugar addition to the substrate under identical conditions, cementing KdoT as a strict KdoIII transferase that does not initiate Kdo addition in *A. baumannii* core OS synthesis.

### GnaT is a UDP-GlcNAcA transferase

With KdoT established as the KdoIII glycosyltransferase, the next logical step was to test whether GnaT could fulfill the remaining UDP-GlcNAcA transferase role. A suitable purified Kdo_3_-lipid IV_A_ or Kdo_3_-lipid A substrate could not be generated in preparative quantities needed to support our *in vitro* assays. To circumvent this, we turned to a coupled assay using the Kdo_2_-[4′-^32^P]lipid IV_A_ substrate as before as well as both CMP-Kdo and UDP-GlcNAcA sugar donors and both KdoT and GnaT enzymes. As we have already proven Kdo_3_-lipid IV_A_ formation *via* KdoT-His_8_ and CMP-Kdo alone, this coupled assay yields *in situ* formation of the appropriate Kdo_3_-[4′-^32^P]lipid IV_A_ substrate needed for GlcNAcA addition, thereby allowing us to attribute any additional substrate modifications to GnaT activity.

**Figure 4:**
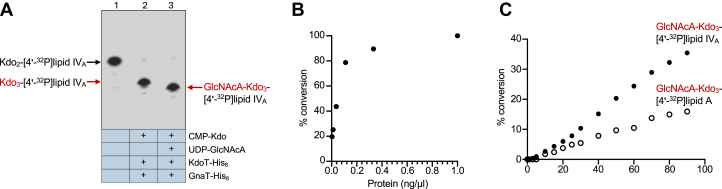
**GnaT is a UDP-GlcNAcA transferase.***A*, TLC of coupled *in vitro* transferase assays. Kdo_2_-[4′-^32^P]lipid IV_A_ (lane 1) is used to generate Kdo_3_-[4′-^32^P]lipid IV_A_ (lane 2) *in situ via* KdoT-His_8_ and CMP-Kdo as demonstrated in Figure 3*B*. This substrate is then converted to GlcNAcA-Kdo_3_-[4′-^32^P]lipid IV_A_ (lane 3) by GnaT-His_8_ and UDP-GlcNAcA, seen as a second downward mobility shift. Data is representative of three independent experiments. *B*, conversion of Kdo_3_-[4′-^32^P]lipid IV_A_ to GlcNAcA-Kdo_3_-[4′-^32^P]lipid IV_A_ as a function of GnaT concentration. Product conversion was quantified *via* TLC densitometry. *C*, conversion of Kdo_2_-[4′-^32^P]lipid IV_A_ to GlcNAcA-Kdo_3_-[4′-^32^P]lipid IV_A_ (*closed circles*) and Kdo_2_-[4′-^32^P]lipid A to GlcNAcA-Kdo_3_-[4′-^32^P]lipid A (*open circles*) over time. Product conversion was quantified *via* TLC densitometry.

As demonstrated in Figure 3, KdoT-mediated transfer of KdoIII to yield Kdo_3_-[4′-^32^P]lipid IV_A_ resulted in a decrease in lipid mobility (Fig. 4*A*, lane 2). We used Kdo_2_-[4′-^32^P]lipid IV_A_ (lane 1) and this Kdo_3_-[4′-^32^P]lipid IV_A_ product (lane 2) as a migration ladder. An additional downward shift relative to the Kdo_3_-[4′-^32^P]lipid IV_A_ product would indicate transfer of an additional sugar. Indeed, when UDP-GlcNAcA was added to the reaction mixture containing CMP-Kdo, KdoT-His_8_, and GnaT-His_8_, the product migrated even further downward (lane 3), consistent with GlcNAcA transfer to Kdo_3_-[4′-^32^P]lipid IV_A_ to form GlcNAcA-Kdo_3_-[4′-^32^P]lipid IV_A_. Similar to our results with KdoT, GnaT was also able to utilize Kdo_2_-lipid A instead of Kdo_2_-lipid IV_A_ (Fig. S3*B*).

We measured the efficiency of GnaT transferase activity under the standard reaction conditions described above. When enzyme concentration was modulated across reactions, the percent of product conversion increased rapidly at lower concentrations (<0.2 ng/μl) and tapered as 100% conversion was approached using concentrations above 0.2 ng/μl (Fig. 4*B*). Calculation of percent conversion of Kdo_3_-lipid IV_A_ to GlcNAcA-Kdo_3_-lipid IV_A_ over time revealed a steadily linear trend (Fig. 4*C*). This time course also showcased a clear preference for the tetra-acylated substrate over hexa-acylated Kdo_2_-lipid A.

### KdoT homologs can be found throughout Acinetobacter and in other Gram-negatives

**Figure 5:**
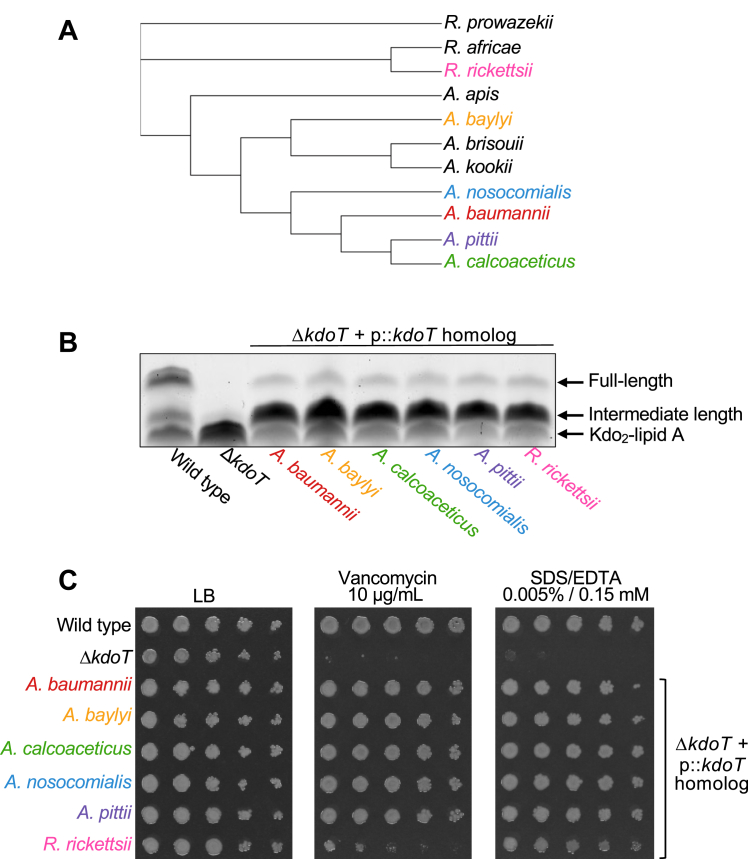
**KdoT homologs are found in other Gram-negative organisms.***A*, phylogenetic tree of select KdoT homologs. Protein sequences were aligned with ClustalW and the resulting Newick tree was visualized in iTOL and rooted using the *Rickettsia* clade as the outgroup. Potential homologs selected for further characterization are labeled with colored branch tips. The tree groups the *Acinetobacter* homologs together with non-*Acinetobacter calcoaceticus-baumannii* (ACB) members branching more distantly, while *Rickettsia* forms a distinct clade. *B*, analysis of LOS patterns of *A. baumannii* Δ*kdoT* expressing *kdoT*-like proteins *in trans.* WT, Δ*kdoT*, and Δ*kdoT* expressing *A. baumannii* KdoT were included as controls. *C*, comparison of antibiotic sensitivity to 10 μg/ml vancomycin or 0.005% SDS + 0.15 mM EDTA with EOP of the strains described in (*B*). EOP and core OS staining results are representative of three biological replicates.

Since KdoT was not previously annotated as a glycosyltransferase and has been labeled as an “ELM1-like mitochondrial fission protein,” we asked whether clear homologs occur outside *A. baumannii*. We focused on KdoT rather than GnaT here because GnaT has already been annotated as a glycosyltransferase and shows high similarity to a multitude of proteins across Gram-negatives, whereas identifying KdoT-like proteins directly tests whether the noncanonical WaaA-independent KdoIII transfer described above extends beyond *A. baumannii*. BLASTp searches against all bacterial proteomes identified numerous KdoT-like proteins across the genus *Acinetobacter* as well as other more distant Gram-negatives such as *Rickettsia*. A phylogenetic tree assembled from select representative species resolved two principal groupings: a Gammaproteobacterial *Acinetobacter* clade and a more distant Alphaproteobacterial *Rickettsia* outgroup [Fig. 5*A*, (32, 33)]. Within *Acinetobacter*, species from the *calcoaceticus-baumannii* (ACB) complex—consisting of *A. baumannii*, *Acinetobacter calcoaceticus*, *A. pittii*, and *A. nosocomialis*—clustered together, whereas species of *A. baylyi* and *A. apis* branched more distantly. Amino acid sequence alignments of each potential homolog can be found in Figure S5. Although detailed core OS structures are not yet available for many of these organisms, the breadth of potential KdoT homologs among different Gram-negative organisms argues that this distinctive mechanism of inner-core Kdo transfer is not unique to *A. baumannii*.

To test whether the identified sequences encoded functional proteins, we expressed readily available homologs (*A. baylyi*, *A. calcoaceticus*, *A. nosocomialis*, *A. pittii*, *R. rickettsii*) from a plasmid in *A. baumannii* Δ*kdoT* and assessed their ability to complement core OS synthesis relative to *A. baumannii*–derived *kdoT*. Detection of LOS by ProQ Emerald staining following SDS-PAGE of whole cell lysates revealed that each homolog mimicked the core OS banding consistently observed for the *kdoT* mutant complemented with the native enzyme, producing a typical pattern of primarily intermediate-length LOS as well as full-length LOS (Fig. 5*B*).

We previously observed that loss of *kdoT* disrupted the outer membrane permeability barrier, resulting in sensitivity to antibiotics that typically do not cross the outer membrane (*e.g.*, vancomycin) and to agents that disrupt OM asymmetry (*e.g.*, SDS-EDTA). In addition to restoring core OS synthesis, we found the identified KdoT homologs could restore outer membrane integrity of Δ*kdoT* (16). Efficiency-of-plating (EOP) assays revealed that while the *kdoT* mutant alone was sensitive to both vancomycin and SDS-EDTA, the complemented strain fully restored resistance (Fig. 5*C*). When Δ*kdoT* was complemented with homologs from other *Acinetobacter* species, rescue approached that observed for the native KdoT complementation. However, when the Rickettsial homolog was expressed, the effect was only partial despite similar restoration of core OS compared to other homologs. This trend was also observed when vancomycin E-tests were used instead of EOP assays (Fig. S6).

Altogether, these data demonstrate that *kdoT* homologs are present across multiple Gram-negative lineages and can substitute for *kdoT in vivo* to promote core OS extension and maintain OM barrier integrity. These findings support a conserved KdoT-dependent mechanism for inner-core KdoIII transfer that departs from the canonical WaaA-only inner core Kdo transfer.

### Predictive modeling of KdoT suggests an alternative mechanism for the inverting GT-B enzyme

**Figure 6:**
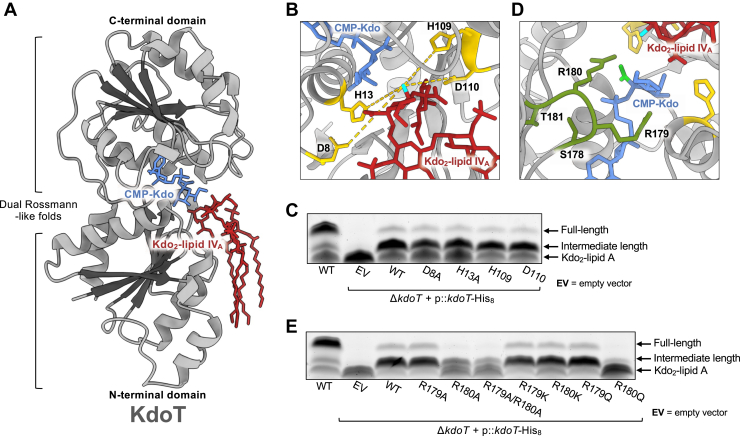
**Predicted structural models of KdoT.** All protein models were predicted with ChaiDiscovery and visualized in ChimeraX v1.10. *A*, KdoT displays GT-B dual Rossmann-like folds and typical ligand-binding characteristics, with N-terminal acceptor binding and C-terminal donor binding. The canonical Rossmannoid ⍺/β/⍺ sandwich is shown in each domain (⍺ = *gray*, β = *black*). *B*, zoomed view of KdoT catalytic cleft. Best candidates for KdoT N-terminal catalytic base residues (*yellow*) and their respective distances from the acceptor hydroxyl of Kdo_2_-lipid IV_A_ (*cyan*) are as follows: D8 (10.6Å), H13 (7.3Å), H109 (8.0Å), and D110 (8.2Å). *Dotted yellow* lines indicate shortest path from the functional group to the acceptor hydroxyl. *C*, analysis of the LOS assembly of Δ*kdoT* expressing KdoT-His_8_ mutants D8A, H13A, H109A, and D110A shows no change in core extension. *D*, two arginine residues belonging to a highly conserved C-terminal SRRT motif (olive) are positioned to interact with the donor Kdo carboxylate (lime), potentially serving as a temporary proton sink. R180 distance from carboxylate is 2.9Å; R179 distance from carboxylate is 8.0Å. *E*, analysis of LOS assembly of Δ*kdoT* expressing the KdoT-His_8_ R180A variant, but not R179A, results in decreased core OS extension. Mutation of both R179 and R180 to an alanine results in a deep truncation chemotype mimicking that of Δ*kdoT*. The R180K variant shows a LOS profile similar to WT, while R180Q results in the partial loss of complementation similar to R180A. Mutation of R179 to a lysine or glutamine shows no change in LOS species. LOS profiles are representative of three biological replicates.

Since this is the first description of KdoT and GnaT enzymatic function in the literature, we sought to further characterize each protein in more detail. We first modeled KdoT using ChaiDiscovery (34) bound to its CMP-Kdo donor and Kdo_2_-lipid IV_A_ acceptor (Fig. 6*A*) This revealed a canonical GT-B architecture with two Rossmann-like ⍺/β/⍺ domains (35, 36) separated by a short linker with the predicted catalytic cleft at the interdomain interface. As expected for GT-B enzymes, the C-terminal domain engages the nucleotide-activated donor (CMP-Kdo, blue) while the N-terminal domain accommodates the lipid-linked acceptor (Kdo_2_-lipid IV_A_, red). Similar docking of each ligand to the expected termini was observed regardless of the degree of acylation of the substrate (data not shown).

Although KdoT is not currently assigned to a characterized glycosyltransferase family, we propose it is an inverting GT-B enzyme based on its use of CMP-β-Kdo in prior *in vitro* assays (Fig. 3) and the resulting ⍺-KdoIII-(2 → 5)-⍺-KdoI linkage in the core OS (15). It has been reported that inverting GT-B enzymes typically use a single catalytic base such as Asp/Glu/His to deprotonate the acceptor for S_N_2-like displacement of the donor leaving group (35, 37). To test whether KdoT follows this canonical scheme, we searched alignments of KdoT homologs for conserved D/E/H residues (Fig. S5) and then examined the model for residues positioned within a permissive ∼10 Å of the acceptor 5-OH. While 10 Å would likely be too distant for nucleophile deprotonation, this generous distance should accommodate any inaccuracies in the predictive model. This analysis provided four candidates—D8, H13, H109, and D110—which cluster in the predicted active site (Fig. 6*B*). Notably, H13 is not conserved outside of the genus *Acinetobacter* based on amino acid alignments but overlays with established catalytic bases in related glycosyltransferases such as the WaaA Kdo transferase of *Aquifex aeolicus* (38, 39) when tertiary structures are superimposed (Fig. S7) We mutated each candidate to alanine in our His_8_-tagged construct and expressed the variants in *A. baumannii* Δ*kdoT*. Surprisingly, these results showed that each mutant retained the ability to restore core OS relative to WT KdoT (Fig. 6*C*), suggesting that KdoT does not rely on any single one of these residues as an essential catalytic base.

Given these results, we considered a noncanonical alternative in which catalysis relies on substrate alignment and electrostatic stabilization by noncatalytic residues (37). In this mechanism, a nearby functional group would stabilize the donor substrate which acts as the catalytic base *in lieu* of an enzyme-derived residue. Returning to the KdoT homolog alignments, we noted a strongly conserved SRRT motif whose arginine residues consistently lie close to the CMP-Kdo donor in predictive models (Fig. 6*D*). We therefore tested their contribution to KdoT activity by alanine substitution and evaluated changes in LOS assembly (Fig. 6*E*). R180A, but not R179A, was unable to fully restore core OS relative to WT KdoT, signaling a decrease in activity and suggesting a more dominant role for R180 in potential substrate positioning in this enzyme. The minor activity retained by the R180A mutant made us wonder whether R179 was able to substitute for the mutation of R180 and continue positioning the CMP-Kdo donor. To test this hypothesis, we mutated both arginine residues of the SRRT motif and evaluated LOS staining patterns. SDS-PAGE revealed that complementation of Δ*kdoT* with this double mutant resulted in a Kdo_2_-lipid A chemotype akin to the R180A single mutant, indicating that R179 is not a suitable substitute for R180 (Fig. 6*E*). Western Blot analysis of whole-cell lysates from each strain confirmed that all mutants maintained overall protein stability, indicating that changes in activity were not due to protein degradation (Fig. S8*A*).

To separate the effects of charge *versus* side-chain geometry of the contributing arginine residues, we generated R179K and R180K mutants. These substitutions maintain positive charge but not the side-chain length and hydrogen-bonding capacity provided by an arginine residue. We also constructed R179Q and R180Q variants, which lack positive charge but roughly preserve structural components (40, 41, 42). LOS analysis showed that the R180K variant fully complemented core OS, indicating full retention of activity, while R180Q showed near complete loss of core OS complementation (Fig. 6*E*). This is consistent with a requirement for positive charge near the nucleotide-activated donor rather than a strict need for arginine geometry. In contrast, R179K and R179Q mutants showed no change in core OS complementation relative to WT KdoT, mimicking R179A results (Fig. 6*E*). Together, the modeling and mutagenesis support an inverting GT-B enzyme in which KdoT promotes Kdo transfer without an essential catalytic base, instead relying on conserved positively charged SRRT residues, especially R180, to electrostatically position and activate the reacting partners.

Together, the modeling and mutagenesis support a possible mechanism in which KdoT functions as an atypical inverting GT-B glycosyltransferase, with the enzyme favoring electrostatic positioning by the conserved SRRT arginine residues rather than the single catalytic base usually required to enable Kdo transfer. Nonetheless, a fully elucidated catalytic mechanism requires further biochemical characterization beyond predictive modeling.

### GnaT displays typical GT-4 family characteristics

**Figure 7:**
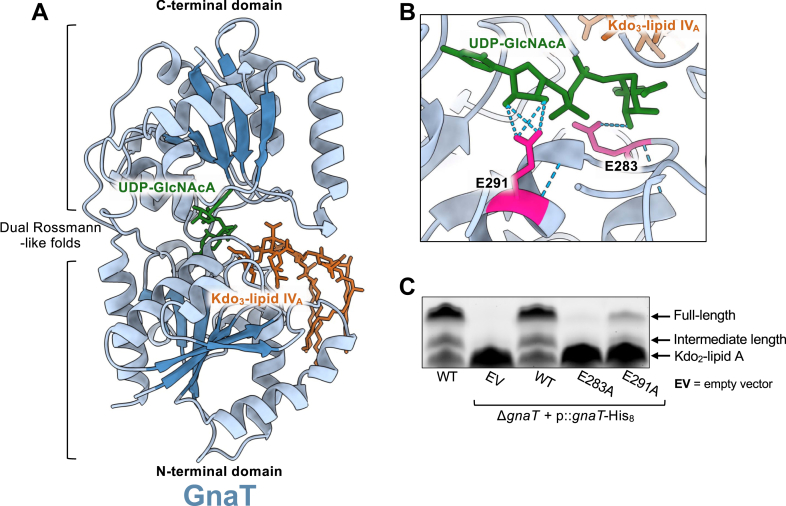
**Structural models of GnaT.** All protein models were predicted with ChaiDiscovery and visualized in ChimeraX v1.10. *A*, GnaT, like KdoT, displays GT-B dual Rossmann-like folds and typical ligand-binding characteristics, with N-terminal acceptor binding and C-terminal donor binding. Kdo_3_-lipid IV_A_ (*orange*) is predicted to bind to the N-terminal domain of GnaT. UDP-GlcNAcA (*green*) is predicted to bind to the N-terminal domain. Rossmannoid ⍺/β/⍺ sandwich is shown in each domain (⍺ = *light blue*, β = *dark blue*). *B*, residues E283 and E291, comprising the Ex7E motif strongly associated with GT-4 family members, are highlighted in *pink*. *Blue* dashed lines indicate available hydrogen bonding sites. *C*, analysis of LOS profiles shows that the E283A mutation results in near-total loss of core OS extension, signaling complete loss of enzymatic activity. However, the E291A mutation results in the partial loss of activity. LOS profiles are representative of three biological replicates.

GnaT, like KdoT, also displays a typical GT-B architecture composed of two Rossmann-like domains (Fig. 7*A*). In the model, the C-terminal domain binds the UDP-GlcNAcA donor (green) while the N-terminal domain binds the Kdo_3_-lipid IV_A_ acceptor (orange). Again, as seen with KdoT, acylation state did not impact whether the acceptor bound to the N terminus (data not shown). Based on amino acid sequence similarity, GnaT falls within the GT-4 glycosyltransferase family, which are retaining enzymes that operate *via* the aforementioned substrate-assisted mechanism in which the UDP leaving group participates in catalysis rather than a dedicated enzymatic general base (43, 44). Since a catalytic base is not expected for this enzyme, we focused on a conserved C-terminal EX_7_E motif that commonly contributes to nucleotide-sugar recognition (45, 46, 47). Assessment of the amino acid sequence revealed that this motif in GnaT is comprised of glutamic acid residues, E283 and E291. Predictive modeling revealed that these amino acids are adequately positioned to contact the UDP-GlcNAcA donor at two distinct positions, with E283 contacting the sugar moiety and E291 contacting the ⍺-phosphate (Fig. 7*B*).

To test the functional contribution of this motif, we mutated each glutamic acid to alanine and assessed the complementation of core OS synthesis by SDS-PAGE of whole-cell lysates in the *gnaT* mutant. Relative to complementation with WT GnaT, both E283A and E291A mutants showed substantially reduced core synthesis with E283 exhibiting a stronger defect (Fig. 7*C*). Immunoblotting confirmed that mutant proteins were expressed comparably to WT, pointing to a true loss of function of catalytic activity rather than protein instability (Fig. S8*B*). In sum, the data suggest that GnaT likely conforms to the GT-4 family enzyme standard in which the conserved C-terminal Ex7E motif supports donor binding.

We had previously shown that both *kdoT* and *gnaT* must be expressed in the cell for sugar transfer to occur (16), although this was demonstrably not the case *in vitro* (Fig. 3). Despite a lack of evidence to support KdoT–GnaT complex formation, we were curious to see whether co-expression of KdoT and the catalytically inactive mutant GnaT_E283A_ could still foster KdoT-mediated sugar transfer. To do so, we co-expressed WT *kdoT* and *gnaT*_E283A_ in a *kdoT*, *gnaT* double mutant which possesses a Kdo_2_–lipid A chemotype. We then analyzed changes in LOS patterns by SDS-PAGE and ProQ Emerald staining as previously described. In comparison to the co-expression of WT *kdoT* and *gnaT*, which restored full-length core oligosaccharide as expected, we found that *kdoT*/*gnaT*_E283A_ co-expression did result in any sugar addition to the Kdo_2_-lipid A chemotype (Fig. S9). This indicates that KdoT is still unable to function in the cell when GnaT is present but inactive despite the availability of possible protein–protein interactions, suggesting an alternative mechanism for their *in vivo* codependence.

## Discussion

This study first and foremost establishes the functions of KdoT and GnaT as CMP-Kdo and UDP-GlcNAcA glycosyltransferases, respectively. In our *in vitro* assays, KdoT alone transferred Kdo to Kdo_2_-lipid IV_A_ while GnaT transferred GlcNAcA to an *in situ*–generated Kdo_3_-lipid IV_A_ substrate. These data define the enzymatic split between KdoIII addition and subsequent GlcNAcA transfer and provide a direct biochemical framework for the early core OS synthesis steps.

Our results represent an alternative to the typical paradigm in which WaaA transfers all inner core Kdo residues. Instead, *A. baumannii* relies on the secondary Kdo transferase KdoT to finish the Kdo trisaccharide. Why evolve this division of labor? One possibility is that KdoT acts as a gatekeeper by dictating the rate of LOS synthesis after initial KdoI and KdoII transfer by WaaA. If nucleotide-activated sugars utilized by diverse cell envelope pathways are limited or downregulated under stress, the cell may conserve resources to synthesize these substrates only when downstream glycosyltransferases or transport machinery are ready. Importantly, however, WaaA synthesis of the baseline Kdo_2_-lipid IV_A_ would remain undisturbed, ensuring a pathway for hepta-acylated lipid A completion and outer membrane integrity.

Evidence for this model comes from the core OS staining pattern of WT *A. baumannii*. Unlike *E. coli* K-12, which assembles a complete core under standard laboratory conditions, *A. baumannii* routinely exhibits three distinct LOS species: a fast-migrating Kdo_2_–lipid A subspecies, LOS with a core OS of intermediate length, and LOS with fully extended core OS (Fig. S1, Figure 5, Figure 7, S9). This distribution of LOS chemotypes in WT *A. baumannii* has also been confirmed by mass spectrometry for multiple strains (16, 48). The coexistence of multiple chemotypes supports a scenario in which WaaA commits to Kdo_2_–lipid A, while subsequent steps are gated by downstream enzymes. Separating KdoIII transfer from WaaA-mediated KdoI/II transfer may provide a physiological advantage by pacing core OS assembly. In summary, decoupling of KdoT from WaaA could provide a unique and adaptable mechanism for balancing efficient core OS synthesis without risking the asymmetric barrier. Additional secondary roles of KdoT in the cellular context—for example, signaling donor availability or crosstalk with other cell machinery—remain intriguing avenues for future study.

The evolutionary reach of this mechanism clearly extends beyond *A. baumannii*. Excitingly, we identified several KdoT homologs across *Acinetobacter* and within *Rickettsia*, although this list is nonexhaustive. Tested KdoT homologs were all able to restore core OS elongation and rescue the outer membrane barrier, with partial rescue for *Rickettsia*, consistent with greater sequence divergence. We suspect this could reflect any number of differences in protein properties including donor utilization, rate of KdoIII transfer to the core OS, membrane context, protein expression and stability, or potentially a secondary KdoT function that is not fulfilled by distant homologs. The presence and similar functional activity of these KdoT-like proteins across *Acinetobacter* and into Alphaproteobacteria, however, argue that the KdoT-dependent KdoIII step is not a strict feature of *A. baumannii* and that this alternative mechanism, different from the traditional WaaA dogma, is shared among diverse Gram-negative organisms.

A notable nuance in our data is the divergence between *in vitro* sufficiency and *in vivo* codependence of *A. baumannii* core assembly. Although KdoT functioned in the absence of GnaT *in vitro*, we previously reported that the two must be co-expressed in the cell to observe transfer of either sugar (16). The inactivity of GnaT without KdoT is expected as its substrate requires KdoT-mediated KdoIII transfer. In contrast, the observation that KdoT does not function without GnaT is less intuitive. We suspect the solo activity of KdoT *in vitro* reflects assay saturation with purified enzyme and freely accessible donor and acceptor, two conditions unavailable in the cell. Importantly, multiple lines of data argue against an obligate KdoT–GnaT protein complex. If such a complex were strictly required, high enzyme concentration alone would be unlikely to restore transfer. Furthermore, immunoprecipitation of KdoT and GnaT did not detect any interacting partners; co-expression of KdoT with GnaT_E283A_ did not allow for KdoT-mediated KdoIII transfer, and AlphaFold3 modeling did not confidently predict a protein–protein interaction. The apparent intracellular codependence may instead reflect less straightforward pathway-level coupling. One possibility is that KdoT is reversible. For example, if GnaT is not available or is unable to attach GlcNAcA to KdoIII, KdoT may recognize and hydrolyze the undecorated KdoIII, returning the acceptor to the Kdo_2_ chemotype. This model is consistent with our previous report that Δ*gnaT* does not accumulate a Kdo_3_-lipid A chemotype (16). Notably, there is precedent for reversibility of enzymes within the core OS pathway—the WaaA reaction itself has been shown to hydrolyze Kdo when incubated with CMP and Kdo_2_-lipid IV_A_ *in vitro,* resulting in Kdo-lipid IV_A_ (18). If KdoT were to follow a similar mechanism, net progress toward core OS synthesis would be contingent on GnaT consuming the Kdo_3_ intermediate by rapidly adding GlcNAcA. Alternatively, an unidentified independent Kdo hydrolase could remove KdoIII when downstream flux is blocked. Kdo hydrolases have been reported in both *Helicobacter pylori* and *Francisella novicida* (31, 49), but no obvious homologs are present in *Acinetobacter* species. Furthermore, characterized Kdo hydrolases act on the periplasmic side of the inner membrane making this possibility unlikely. Still, either scenario could explain the apparent in-cell codependence without requiring a KdoT–GnaT protein complex.

Our modeling and mutagenesis did not reveal a single essential catalytic base for KdoT, an unusual outcome for an inverting GT-B enzyme. While we hesitate to over-interpret predictive models, the combined data are consistent with a mechanism in which conserved arginine residues within a SRRT motif provide electrostatic positioning of the donor and acceptor rather than classical general base catalysis. A similar mechanism has been proposed in two inverting GT-B fucosyltransferases, FUT1 from *Arabidopsis thaliana* and POFUT1 from *Caenorhabditis elegans* (50, 51). These studies similarly failed to identify a general base and proposed instead that the β-phosphate of the nucleotide sugar donors, supported by conserved arginine residues, may participate in proton transfer instead; however, no definitive mechanism was established for either. We view our results as hypothesis-generating and anticipate that additional biochemistry will be required to resolve whether KdoT truly operates without a protein-based general base.

In the case of GnaT, we found that our structural modeling supports amino acid similarity-based characterization of the protein as a retaining GT-4 glycosyltransferase which does not rely on an amino acid side chain as a general base (43, 44). Consistent with precedent, catalysis likely proceeds by a substrate-assisted mechanism in which the contacts made by the acceptor and donor within the catalytic cleft achieve proper substrate positioning, thus allowing for deprotonation of the acceptor hydroxyl by a nonprotein moiety such as the β-phosphate of the UDP-donor (37). As expected, the canonical EX_7_E motif is embedded in the donor pocket, and our mutational analysis reduced complementation without altering protein expression, indicating a role in donor recognition and positioning. Taken together, the predictive modeling, mutagenesis, and LOS structural data are compatible with a substrate-assisted retaining mechanism for GnaT.

Together, our results define an unusual early core OS synthesis pathway in *A. baumannii* in which KdoT catalyzes the addition of KdoIII and GnaT follows *via* transfer of GlcNAcA, providing a concrete order of reactions for inner core construction in conjunction with our previous data (16). This separation of KdoIII transfer from KdoI/II addition represents a clear departure from the canonical WaaA-only model of complete inner core Kdo addition *via* a multifunctional enzyme. The presence of KdoT homologs across unique Gram-negative taxa further implies that this organization of inner core OS synthesis is not confined solely to *A. baumannii*. Further exploration in these organisms may provide necessary context for the mechanism. Viewed together, these findings outline a novel method of core OS assembly that may help explain how early core OS synthesis is coordinated with LOS and outer membrane biogenesis.

## Experimental procedures

### Bacterial strains and growth

Strains used in this study are found in Table S1. All *E. coli* and *A. baumannii* cultures were routinely grown in LB at 37 °C with shaking. Cultures were supplemented with 30 μg/ml kanamycin when pMMB was present, 100 μg/ml ampicillin when pET21 was present, and 34 μg/ml chloramphenicol when pLysS was present.

### Cloning

All plasmids and primers used in this study are found in Table S1. Plasmids were purified using the QIAprep Spin Miniprep Kit (Qiagen). DNA fragments used in cloning procedures were amplified using TaKaRa ExTaq polymerase (TaKaRa Bio Inc.) and purified using the QIAquick PCR Purification Kit (Qiagen). Restriction enzyme cut sites, indicated in primer names, were added during fragment amplification. Inserts and vectors were digested with applicable restriction enzymes followed by vector treatment with Antarctic phosphatase. Inserts and vectors were ligated together and transformed into DH5⍺ or XL1-blue. Restriction enzymes, Antarctic phosphatase, and T4 DNA ligase were obtained from NEB. *kdoT* homolog sequences from *A. baylyi*, *A. pittii*, and *R. rickettsii* were synthesized by GenScript and subcloned into pMMB. All constructs were verified by whole plasmid sequencing *via* Plasmidsaurus.

Amino acid substitutions for site-directed mutagenesis were generated using PFU Turbo Polymerase AD (Agilent). Briefly, point mutations were introduced to the templates pMMB::*kdoT*-His_8_ or::*gnaT*-His_8_ *via* PCR with primers that encoded the desired mutation. Following amplification, remaining template was degraded *via* DpnI digest. Plasmids were purified using the QIAquick PCR Purification kit (Qiagen), transformed into DH5⍺, miniprepped, and screened for the correct mutation *via* whole plasmid sequencing (Plasmidsaurus).

### Overexpression and purification of KdoT and GnaT

Overnight *E. coli* BLR(DE3) pLysS pET21a::*kdoT-His*_*8*_ or::*gnaT-His*_*8*_ cultures were diluted 1:100 into LB supplemented with 34 μg/ml chloramphenicol and 100 μg/ml ampicillin. Cultures were grown at 37 °C with shaking until an OD_600_ of ∼0.6 was reached. Protein expression was induced with 1 mM IPTG and continued for 4 more hours at 37 °C before harvesting cells by centrifugation (10,000*g* for 10 min). All cultures were washed once with PBS and frozen at −80 °C. To collect membrane fractions, pellets were resuspended in buffer consisting of 50 mM Hepes pH 7.5, 0.5 M NaCl, and 20 mM imidazole to a final concentration of 200 mg/ml. 1 mg/ml lysozyme and 20 units/ml benzonase were added before single passage through a mechanical cell press at 20,000 psi. Unbroken cells were removed by centrifugation (10,000*g,* 10 min, 4 °C) and the remaining supernatant (cell-free extract) was separated into membrane and cytosol fractions by ultracentrifugation (100,000*g*, 1 h, 4 °C). Membranes were resuspended in buffer as above and proteins were solubilized in the presence of 1% Triton X-100 with consistent rotation for 2 h at 4 °C. Samples were centrifuged (17,000*g*, 10 min, 4 °C) to remove remaining membrane debris, and the supernatant was stored at −80 °C.

To purify solubilized proteins, samples were first run through 0.45 μM filters (Whatman) to remove debris resulting from freeze-thaw cycles and loaded on a 1 ml HisTrap FF Ni^2+^-Sepharose Column (Cytiva) that had been pre-equilibrated with five column volumes of water followed by five column volumes of wash buffer (50 mM Hepes pH 7.5, 0.5 M NaCl, 20 mM imidazole, 0.05% Triton X-100). After loading the sample, the column was washed with 10 column volumes of wash buffer. Elution was performed with a linear (0–100%) gradient of elution buffer (50 mM Hepes pH 7.5, 0.5 M NaCl, 500 mM imidazole, 0.05% Triton X-100, 10% glycerol) over 20 column volumes with a fraction size of 0.5 ml each. Ten microliters of each fraction was loaded to SDS-PAGE followed by Coomassie staining as previously described to determine protein purity, and the resulting data was used to combine fractions containing only the protein of interest. The eluted protein was concentrated and buffer exchanged into a buffer lacking imidazole (50 mM Hepes, 100–250 mM NaCl, 0.05% Triton, 10% glycerol) using a Vivaspin 10 kDa-cutoff spin column (Cytiva). KdoT-His_8_ exchange buffer contained 250 mM NaCl while GnaT-His_8_ exchange buffer contained 100 mM NaCl. Bicinchoninic acid (BCA) assays were performed using the Pierce BCA Protein Assay Kit (ThermoScientific) to establish final protein concentration.

### Immunoprecipitation

Overnight *A. baumannii* cultures were diluted 1:100 into 50 ml LB broth supplemented with 30 μg/ml kanamycin and 1 mM IPTG and grown at 37 °C with shaking until all cultures reached an OD_600_ of 1.0. Cultures were split into two 25-mL aliquots, centrifuged at 5000*g* for 10 min at 4 °C, and washed once with PBS. One 25-mL aliquot from each culture was washed again with PBS and resuspended in 2 mM ethylene glycol *bis*(succinimidyl succinate) (Thermo Fisher Scientific) crosslinker to stabilize weak or transient protein–protein interactions. Resuspensions were rotated for 30 min at room temperature, and the crosslinking reaction was quenched *via* addition of 5 μl 1M Tris, pH 7.5, for 15 min with rotating at room temperature. Samples were centrifuged at 17,000*g* for 1 min and washed with 1 ml of 50 mM Tris pH 7.5. Pellets were frozen at −80 °C. Pellets were then thawed, resuspended in 1 ml lysis buffer (1X BugBuster, 0.5 mg/ml lysozyme, 1X benzonase, 1 cOmplete mini EDTA-free protease inhibitor cocktail tablet per 10 ml lysis buffer), and rotated at room temperature for 1 h. Lysates were centrifuged at 17,000*g* for 10 min and the supernatant was transferred to a new tube. Immunoprecipitation was conducted *via* the Pierce Magnetic HA-Tag IP/CoIP Kit (Thermo Fisher Scientific) and proteins eluted in 50 μl elution buffer. Lysate inputs and immunoprecipitation eluents were used for the analysis of protein–protein interaction.

### SDS-PAGE and Western immunoblotting

LB broth was inoculated with a single colony and incubated at 37 °C overnight. Hundred micromolars of IPTG was used for induction where necessary. For identification of protein localization and purification as shown in Figure 2, whole-cell lysate was generated *via* mechanical cell press as described above. Soluble- or membrane-only fractions were similarly isolated *via* ultracentrifugation as described above. To detect general protein expression as shown in Figure S8, whole-cell lysate was generated by pelleting 1 ml of culture at 17,000 x *g* for 1 min, resuspending in 1% SDS, and boiling for 10 min at 100 °C. Samples were cooled to room temperature and centrifuged at 17,000*g* for 10 min. In all experiments except for immunoprecipitations, protein concentration was quantified by BCA assay and normalized based on protein concentration *via* dilution in LDS sample buffer (Invitrogen) + 5% β-mercaptoethanol prior to loading to NuPAGE 10% Bis-Tris gels (Invitrogen) in equal volumes. Gels were run at 100V for 2 h. Proteins were transferred to 0.45 μm nitrocellulose membranes (Amersham) in the presence of Towbin buffer using the Trans-Blot SD semidry transfer cell (Bio-Rad). All blots were blocked for 1 hour in a 2% milk phosphate-buffered saline + 0.1% Tween-20 (w/v) (PBST) solution followed by incubation overnight with primary mouse or rabbit ⍺-His antibody (GenScript) as specified, washed with PBST, and incubated with the corresponding secondary Cy5 goat anti-mouse or anti-rabbit IgG for 1 h (Invitrogen). Blots were washed with PBST to remove unbound antibody and imaged with the ChemiDoc MP imaging system (Bio-Rad)

### LOS staining

LB broth was inoculated with a single colony and incubated at 37 °C overnight. Hundred micromolars of IPTG was added for induction where necessary. Whole cell lysates were prepared and stained for LOS as previously described (16).

### Preparation of radiolabeled substrates

[4′-^32^P]lipid IV_A_ was prepared as previously described (28, 52) using 100 μCi of [γ-^32^P]ATP (Revvity Health Sciences Inc.), tetraacyldisaccharide 1-phosphate, and *E. coli* BLR(DE3) pLysS pJK2 membranes overproducing the 4′-kinase LpxK. This substrate was then used to prepare Kdo_2_-[4′-^32^P]lipid IV_A_ *via* addition of CTP disodium salt (Sigma-Aldrich), Kdo ammonium salt (Sigma-Aldrich), purified CMP-Kdo synthetase KdsB, and purified bifunctional *E. coli* Kdo transferase WaaA. To generate hexa-acylated Kdo_2_-[4′-^32^P]lipid A, lipid A late acyltransferases LpxL and LpxM were used to further acylate Kdo_2_-[4′-^32^P]lipid IV_A_ as previously described (30, 31) with acyl-ACPs serving as the fatty acyl donors.

### *In vitro* assay of KdoT and GnaT enzymatic activity

Assays were conducted under optimized conditions in 10 μl aliquots containing 50 mM Hepes pH 7.5, 10 mM MgCl_2_, 0.05% Triton X-100, 10 μM Kdo_2_-[4′-^32^P]-lipid IV_A_ or Kdo_2_-[4′-^32^P]-lipid A (5000 CPM), 5 ng/ml of KdoT-His_8_ and/or GnaT-His_8_ when present, 1 mM UDP-GlcNAcA (Creative BioLabs), and an estimated 1 mM CMP-Kdo. The CMP-Kdo concentration is approximate due to its generation *in situ via* 10 mM CTP, 4 mM Kdo, 10 mM MgCl_2_, and 0.3 mg/ml KdsB. This reaction was allowed to proceed for 10 min at room temperature prior to its addition to *in vitro* assays in which KdsB is expected to remain active and continue production of CMP-Kdo. *In vitro* assays were incubated at 37 °C for 30 min.

For assays measuring the effect of enzyme concentration on substrate conversion, individual reactions were conducted under the conditions described above. Reactions measuring KdoT-catalyzed KdoIII transfer used enzyme concentrations ranging from 0.012 ng/ml to 1 ng/ml. Coupled reactions measuring LpsB-catalyzed GlcNAcA transfer held KdoT concentration at 10 ng/ml and modulated LpsB concentration from 0.004 ng/ml to 1.0 ng/ml. For assays measuring enzyme activity over time, reactions were conducted under the conditions described above, but with 0.1 ng/ml enzyme to ensure detection within the linear range.

All reactions were stopped by spotting 5 μl aliquots to a Silica Gel 60 thin layer chromatography plate (Merck), air drying, and developing in a chloroform/pyridine/88% formic acid/water (30:70:16:10 v/v/v/v) solvent system. If [4′-^32^P]-lipid IV_A_ was used as the substrate, a solvent ratio of 50:50:16:5 v/v/v/v was used instead. Plates were then air dried, exposed to a phosphorimaging screen overnight, and imaged on a Typhoon NIR Plus (Amersham).

### Bioinformatic analysis

The *A. baumannii* ATCC 17978 KdoT protein sequence was queried against the NCBI nonredundant database using BLASTp and all available and complete homologs returned by the search were collected. Exact duplicates and truncations/partial proteins were excluded. Representatives from *Acinetobacter* and *Rickettsia* genera only were selected from this list for further phylogenetic analysis. Amino acid sequences were aligned with ClustalW v1.2.4 (32) using default parameters. The resulting alignment was used to generate a neighbor-joining phylogram *via* ClustalW and the result was exported in Newick format for tree building and visualization with iTOL v7.2.1 (33). The tree was rooted using the *Rickettsia* clade as the outgroup. Tip labels were manually color-coded by species to indicate homologs selected for further analysis.

### EOP assays

EOP assays were conducted as previously described (16). Overnight cultures were standardized based on OD_600_ measurements and serially diluted in a 96-well plate in LB broth using a multichannel pipette. The cultures were then replica plated onto LB agar media containing no antibiotic, 10 μg/ml vancomycin, or 0.005% SDS + 0.15 mM EDTA and incubated overnight at 37 °C. All LB media contained 100 μM IPTG for plasmid induction. Images were taken using a UVP ColonyDoc-It (Analytik Jena).

### MIC measurements

Minimal inhibitory concentrations (MICs) were measured *via* E-strip (BioMerieux). Overnight cultures were back-diluted 1:100 and grown to an OD_600_ of 0.5 to 0.6 in the presence of 30 μg/ml kanamycin and 100 μM IPTG where necessary. Cultures were spread on LB agar plates and dried. Sterile E-strips were placed in the center of each plate and incubated overnight at 37 °C. The MIC was defined as the point where the zone of growth inhibition intersected the E-strip gradient.

### Protein modeling

The amino acid sequences of KdoT and GnaT from *A. baumannii* ATCC 17978 were submitted to ChaiDiscovery (34) for structure prediction. Multiple sequence alignments were generated by the service using MMseqs2 with default parameters. Unless otherwise noted, default ChaiDiscovery settings were used, and no restraints were applied. For KdoT, the acceptor modeled was Kdo_2_-lipid IV_A_ and the donor was CMP-Kdo. For GnaT, the acceptor was Kdo_3_-lipid IV_A_ and the donor is UDP-GlcNAcA. Ligands were drawn in ChemDraw and SMILES strings were supplied to ChaiDiscovery for placement during complex prediction. No covalent linkages or positional restraints were specified. Ligand identities were verified visually using ChimeraX-1.10 (53) to ensure complete structures with logical stereochemistry. For each structure prediction, ChaiDiscovery returned ranked models. The top-ranked complex based on the default ranking metric was exported without additional refinement. Per-residue confidence metrics for multiple models were inspected to confirm consistent domain folds in the N- and C-terminal domains for each protein.

UCSF ChimeraX-1.10 was used for figure preparation and proteins and ligands were colored for clarity. Interatomic distances between noted atoms were measured with the ChimeraX distance tool. Hydrogen bonds were identified for indicated residues with the H-bonds tool using default geometric criteria. No manual docking or energy minimization was performed post-export. Predicted complexes were used qualitatively to visualize domain organization, donor and acceptor docking, and proximity of conserved residues in the putative catalytic cleft. The models were not used to derive kinetic parameters or to assert a definitive catalytic mechanism.

## Data availability

Where appropriate, original data images and replicate data have been deposited to the open repository Zenodo (see https://zenodo.org/records/17866643).

## Supporting information

This article contains supporting information (16).

## Conflicts of interest

The authors declare that they have no conflicts of interests with the contents of this article.
