# Supplementary material for: Noncanonical lipooligosaccharide assembly in Acinetobacter baumannii is mediated by the glycosyltransferases KdoT and GnaT
Source: J Biol Chem. 2025 Dec 23;302(2):111103. doi: 10.1016/j.jbc.2025.111103 (PMC12856342; doi:10.1016/j.jbc.2025.111103)
Supplement: Supporting information [file mmc1.pdf]

Supporting information for "**Noncanonical lipooligosaccharide assembly in *Acinetobacter baumannii* is mediated by the glycosyltransferases KdoT and GnaT**" by VanOtterloo and co-workers:

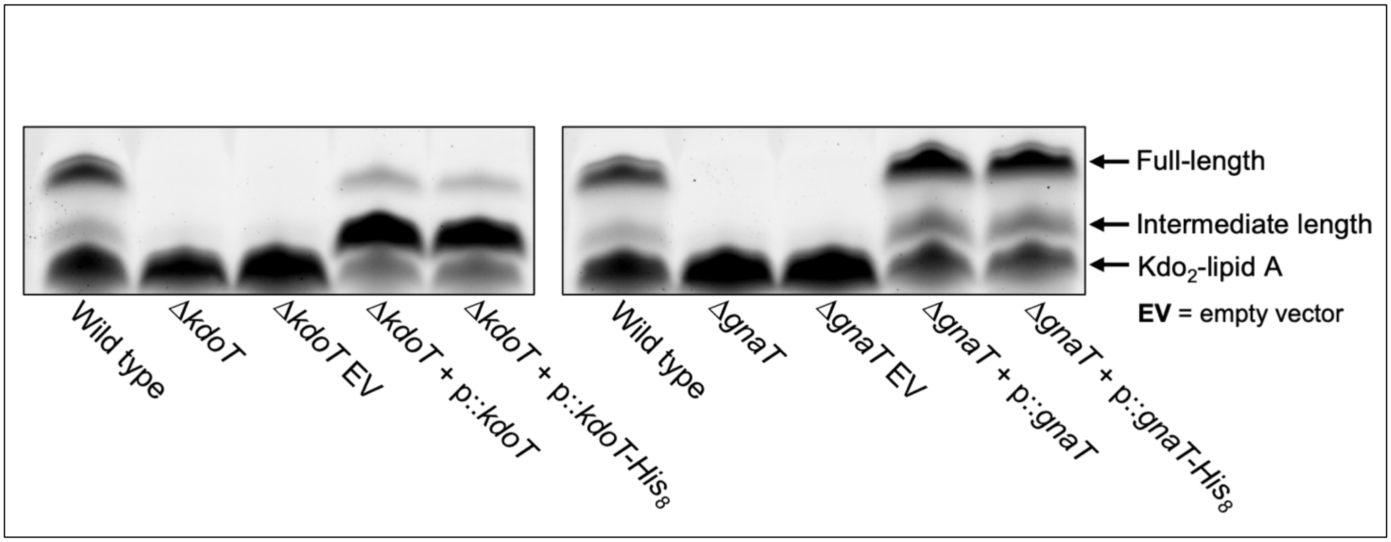

Figure S1: Histidine-tagged KdoT and GnaT are functional relative to untagged proteins. SDS-PAGE and core OS staining of *A. baumannii* proteinase K-treated whole cell lysates. Wild type displays typical staining pattern with three major LOS species: Kdo_2-lipid A, LOS of intermediate length, and full-length LOS chemotypes. In contrast, Δ*kdoT* and Δ*gnaT* are fully truncated to Kdo_2-lipid A. Complementation of Δ*kdoT* with either tagged or untagged *kdoT* results in partial restoration to full-length core OS with primarily intermediate length chemotype. Complementation of Δ*gnaT* with either tagged or untagged *gnaT* results in the production of wild-type LOS species. Results are representative of three biological replicates.

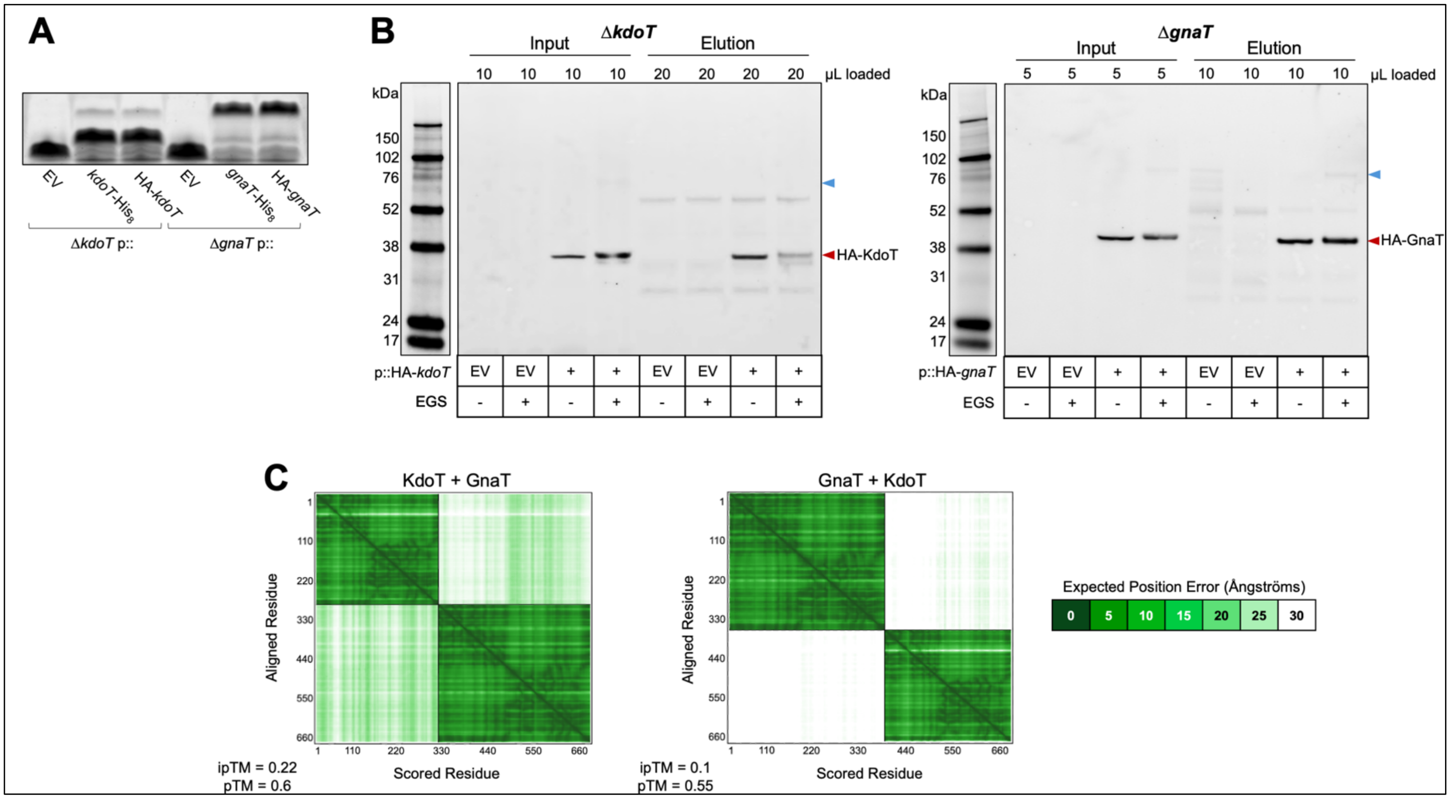

Figure S2. Protein-protein interactions are not detected between KdoT and GnaT. (A) SDS-PAGE and core OS staining of *A. baumannii* proteinase K-treated whole cell lysates. N-terminal HA-tagged constructs complement Δ*kdoT* and Δ*gnaT* core OS synthesis similar to C-terminal His-tagged constructs. Data are representative of two biological replicates. **(B)** Whole-cell lysates from Δ*kdoT* p::HA-*kdoT* and Δ*gnaT* p::HA-*gnaT* strains, as well as their empty vector controls (EV), were either untreated or crosslinked with EGS prior to immunoprecipitation. Successful crosslinking is indicated by an upward mobility shift induced by intramolecular crosslinking. Input lysates and corresponding elution fractions were probed by immunoblotting with anti-HA antibody. The expected HA-tagged species are indicated by red arrowheads. Bands at ~67 kDa or ~82 kDa (blue arrows) are predicted to indicate KdoT and GnaT dimers, respectively, while a protein-protein complex is expected to migrate at ~75 kDa. Data are representative of three biological replicates. **(C)** Predicted alignment error (PAE) plots for potential protein-protein interactions between KdoT and GnaT (left) or GnaT and KdoT (right) generated using AlphaFold3. The order of proteins indicates the order in which amino acid sequences were provided. The accompanying expected position error bar indicates predicted alignment error in Å, with dark green representing lower PAE values.

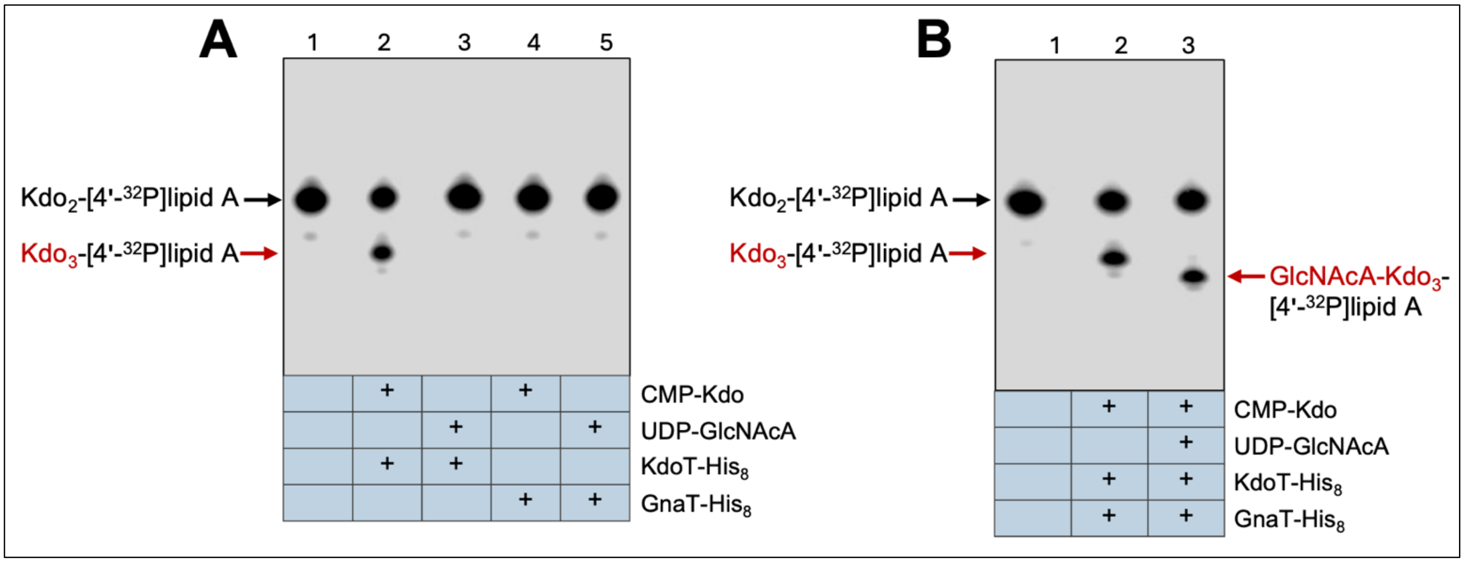

Figure S3: Hexa-acylated Kdo_2-lipid A is a suitable substrate for KdoT-mediated KdoIII and GnaT-mediated GlcNAcA transfer. TLC of *in vitro* assays showing KdoIII transfer to Kdo_2-[4'-^{32}P]lipid A **(A)** or GlcNAcA transfer to Kdo_3-[4'-^{32}P]lipid A via coupled assay **(B)** as described in **Fig. 4**. Data are representative of three biological replicates.

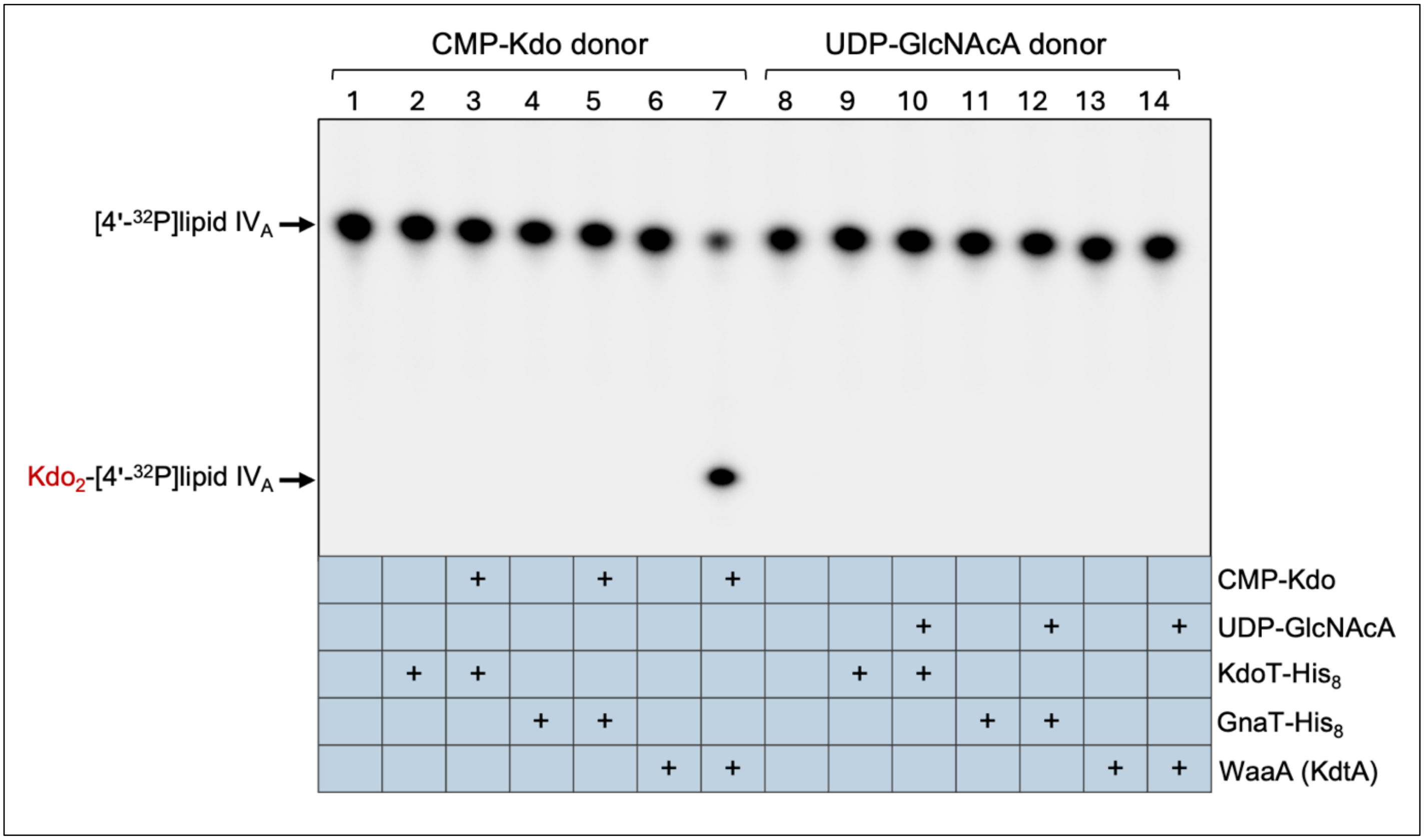

Figure S4: KdoT and GnaT cannot transfer sugars to lipid IV_A. TLC of *in vitro* assays using [4'-^{32}P]lipid IV_A (lane 1, lane 8) as the acceptor substrate in lieu of Kdo_2-[4'-^{32}P]lipid IV_A. Either CMP-Kdo or UDP-GlcNAcA was used as the donor. Only the bifunctional *E. coli* Kdo transferase WaaA was able to transfer Kdo to [4'-^{32}P]lipid IV_A resulting in Kdo_2-[4'-^{32}P] lipid IV_A (lane 7). No activity was seen when KdoT or GnaT was used. Data are representative of three biological replicates.

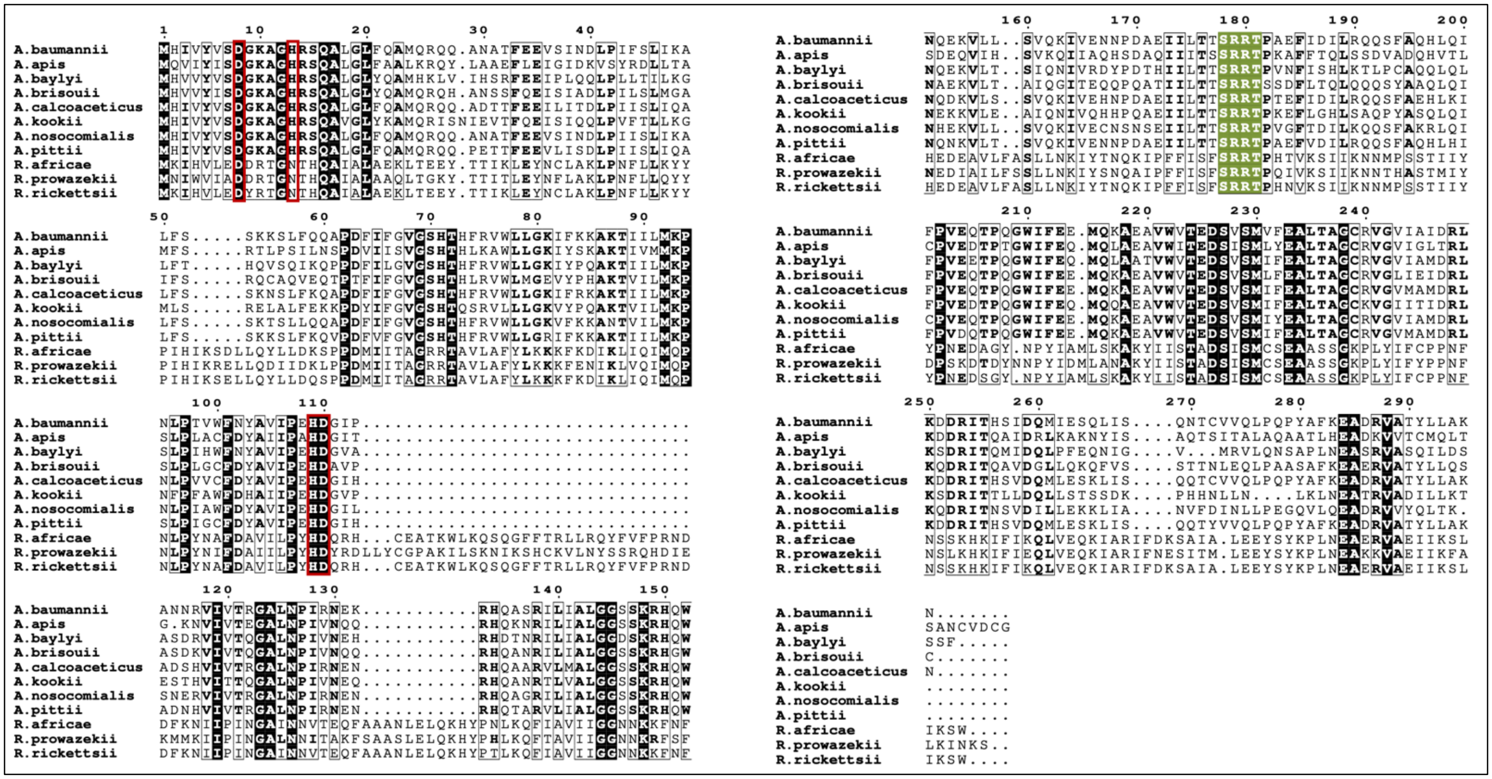

Figure S5. Multiple sequence alignment of KdoT-family homologs reveals an invariant SRRT motif. Protein sequences of KdoT from *A. baumannii* and representative homologs from several Gram-negative bacteria were aligned with ClustalW. The resulting sequence alignment was rendered in ESPript 3.x using the %Equivalent color scheme. Positions are numbered relative to the *A. baumannii* KdoT sequence and gaps are shown as dashes. Residues identical across all sequences appear in reverse contrast and chemically similar positions are shaded according to ESPript %Strict: Residues inside black outlined boxes reach the %Strict threshold but are not invariant. Within these boxes, residues identical to the consensus sequence are bolded. A conserved SRRT motif (Ser-Arg-Arg-Thr) is present in every homolog (shaded in green), highlighting it’s a potential functional importance within this glycosyltransferase family. Other residues of interest are highlighted by a red box.

	Strain/Homolog	Vancomycin (µg/ml)
	Wild Type	96 ± 0
	Δ*kdoT*	2 ± 1
Δ*kdoT* + p::*kdoT* homolog	*A. baumannii*	117 ± 19
	A. baylyi	117 ± 19
	A. calcoaceticus	117 ± 19
	A. nosocomialis	149 ± 37
	A. pittii	149 ± 37
	R. rickettsii	7 ± 1

Figure S6. Vancomycin susceptibility of Δ*kdoT* strains complemented with KdoT homologs. Vancomycin E-tests (BioMerieux) were performed on wild type, Δ*kdoT*, and Δ*kdoT* complemented with KdoT homologs from *A. baumannii* or the indicated species. MIC values are shown as the mean ± standard deviation from three biological replicates.

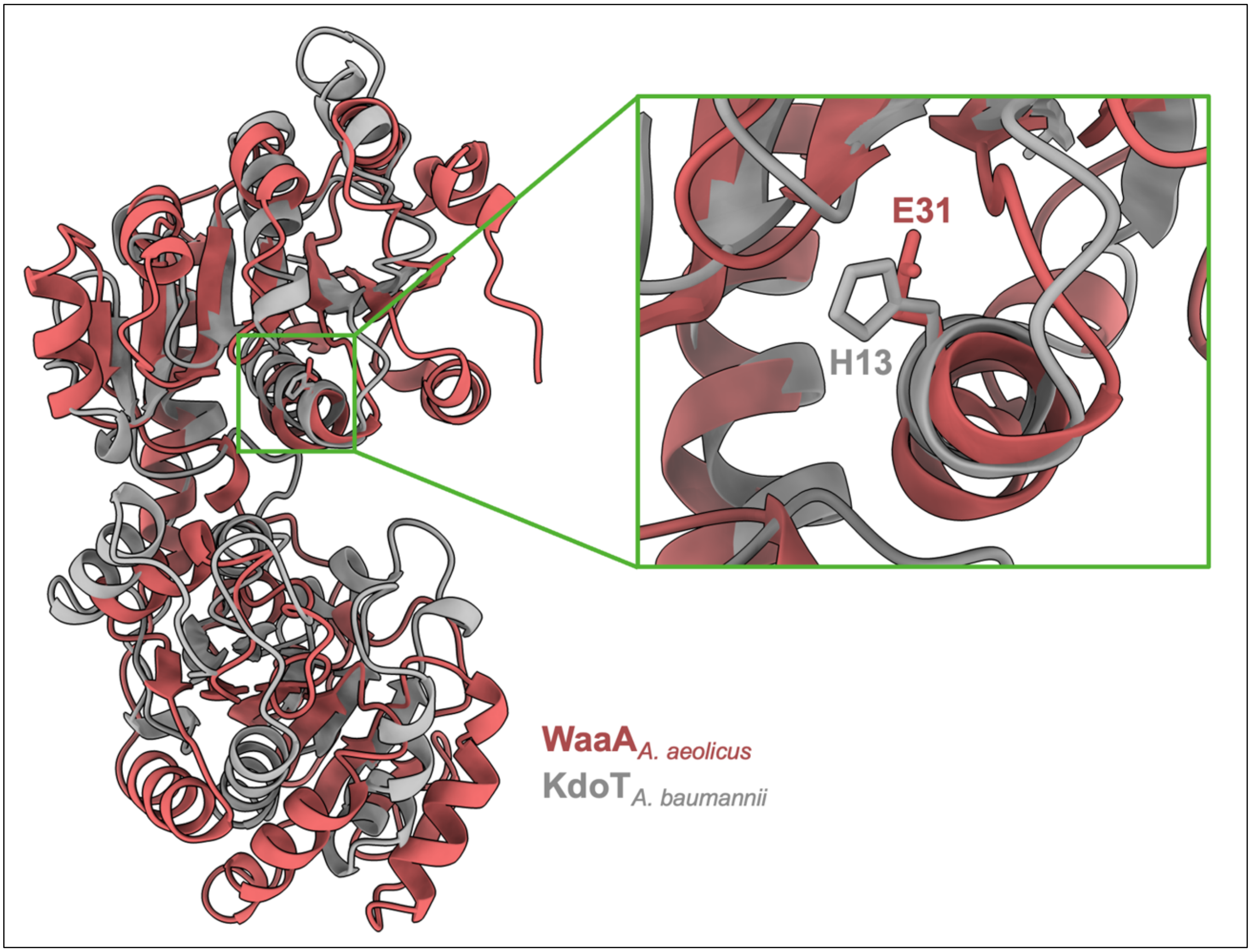

Figure S7. Structural overlay with *Aquifex aeolicus* WaaA highlights KdoT His13 as a possible catalytic base. KdoT model was predicted with ChaiDiscovery and visualized in ChimeraX v1.10. Structure of *Aquifex aeolicus* WaaA was accessed via PDB (2XCI). Both structures were aligned via ChimeraX Matchmaker function (RMSD between 19 pruned atom pairs = 1.359 Å). Box highlighted in green depicts magnified view of confirmed *A. aeolicus* WaaA catalytic base E31 overlapping with *A. baumannii* KdoT catalytic base candidate H13.

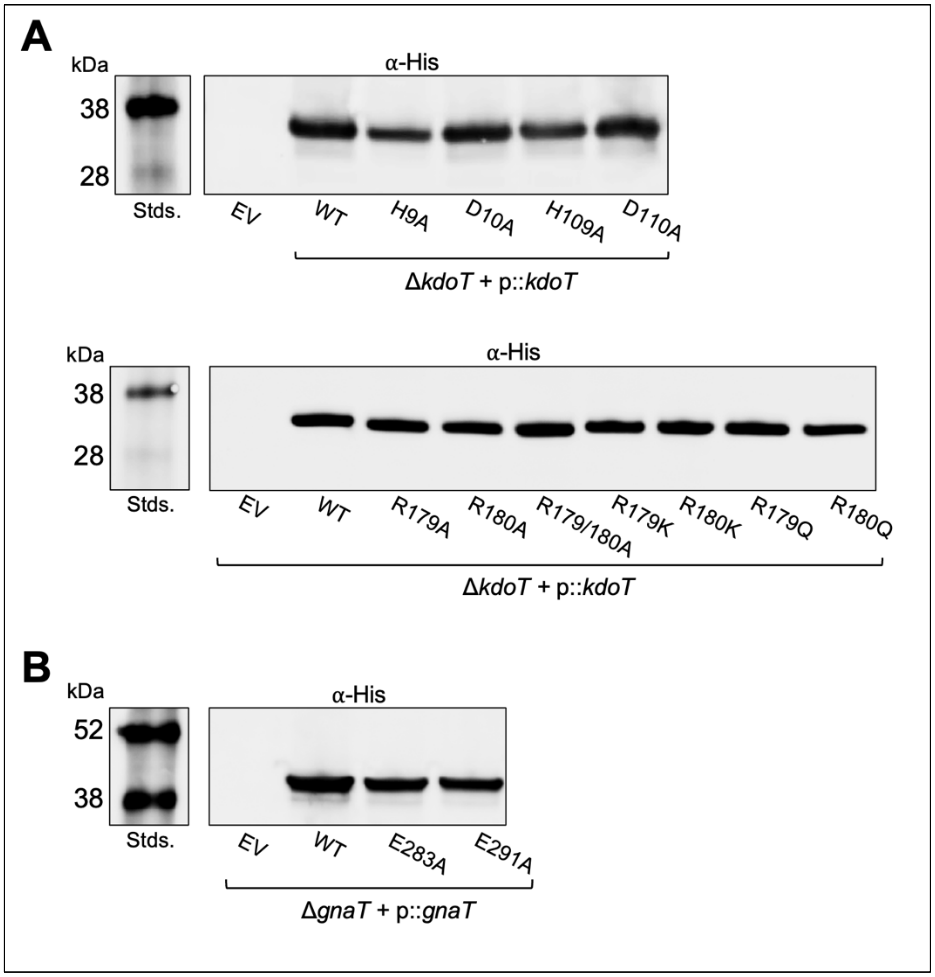

Figure S8. Immunoblotting of whole-cell lysate from *A. baumannii* expressing site-directed KdoT and GnaT His-tagged mutants. (A) Anti-histidine immunoblots of *A. baumannii* Δ*kdoT* expressing various KdoT-His_8 variants. ***Top:*** Immunoblot of Δ*kdoT* p::*kdoT* expressing potential enzyme-derived catalytic base mutants evaluated in **Fig. 6B and C**. ***Bottom:*** Immunoblot of Δ*kdoT* p::*kdoT* expressing SRRT motif arginine mutants evaluated in **Fig. 6D and E**. **(B)** Anti-histidine immunoblot of *A. baumannii* Δ*gnaT* expressing GnaT-His_8 variants E283A and E291A evaluated in **Fig. 7C.** All results are representative of three biological replicates.

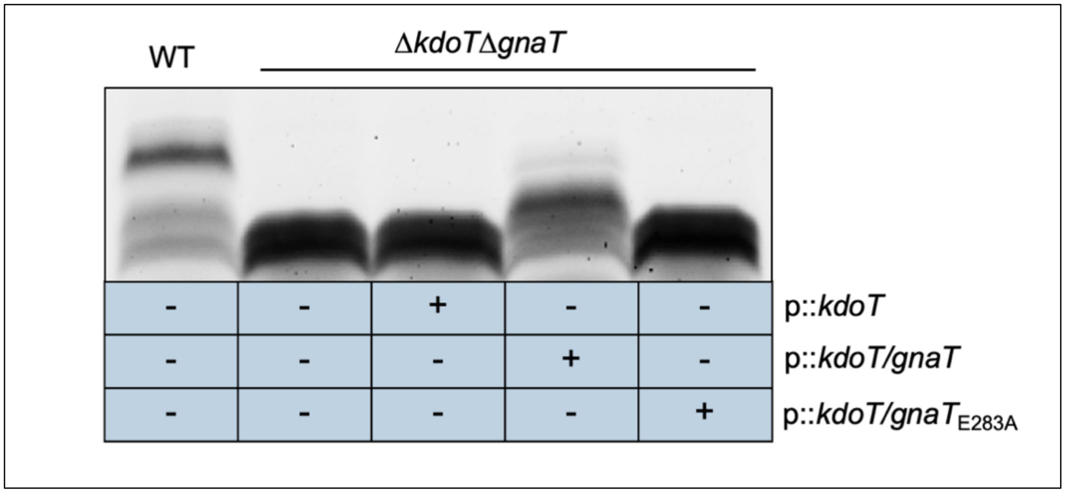

Figure S9. Co-expression of KdoT and catalytically inactive $GnaT_{E283A}$. Whole-cell lysates of indicated *A. baumannii* strains were treated with proteinase K, separated by 16% Tricine SDS-PAGE, and stained for core OS. *ΔkdoTΔgnaT* displays a Kdo_2-lipid A chemotype which is unchanged by expression of KdoT or KdoT/$GnaT_{E283A}$ expression. Co-expression of wild type KdoT and GnaT results in primarily intermediate-length core OS as well as some full-length, which is typical for strains overexpressing KdoT.

Table S1: Strains, Plasmids, and Primers used in this study.

Strain	Genotype	Reference
A. baumannii 17978	Wild type 17978 UN, isogenic parent of all 17978 strains used	ATCC
A. baumannii 17978	Δ*kdoT* (*A1S_2903)*	(1)
A. baumannii 17978	Δ*gnaT* (*A1S_0430, lpsB*)	(1)
A. baumannii 17978	Δ*kdoT*Δ*gnaT*	(1)
E. coli BLR (DE3) pLysS	F^-*ompT hsdS*$_B$(r_B^- m_B^-) *gal lac ile dcm* Δ(*srl-recA*) *i306*::Tn*10* (tet^R)(DE3). pLysS plasmid carries T7 lysozyme gene and CmR cassette.	Novagen
**Plasmid**	**Description**	**Reference**
pMMB	pMMB67EH. IPTG-inducible vector with KanR cassette	(58)
pMMB::*kdoT*	pMMB67EH with *kdoT*	(1)
pMMB::*gnaT*	pMMB67EH with *gnaT*	(1)
pMMB::*kdoT-His*$_8$	pMMB67EH with C-terminally His$_8$-tagged *kdoT*. Construct was synthesized by GenScript	This study, GenScript
pMMB::*gnaT-His*$_8$	pMMB67EH with C-terminally His$_8$-tagged *gnaT*. Construct was synthesized by GenScript	This study, GenScript
pET21a(+)	IPTG-inducible expression vector with AmpR cassette	Novagen
pET21a::*kdoT*-His$_8$	pET21a(+) with C-terminally His$_8$-tagged *kdoT*	This study
pET21a::*gnaT*-His$_8$	pET21a(+) with C-terminally His$_8$-tagged *gnaT*	This study
pMMB::*kdoT* (*A. baylyi*)	pMMB67EH with *kdoT* homolog from *A. baylyi* ADP1. Synthesized by GenScript	This study, GenScript
pMMB::*kdoT* (*A. calcoaceticus*)	pMMB67EH with *kdoT* homolog from *A. calcoaceticus* 1217	This study
pMMB::*kdoT* (*A. nosocomialis*)	pMMB67EH with *kdoT* homolog from *A. nosocomialis* M2	This study
pMMB::*kdoT* (*A. pittii*)	pMMB67EH with *kdoT* homolog from *A. pittii* ST220. Synthesized by GenScript	This study, GenScript
pMMB::*kdoT* (*R. rickettsii*)	pMMB67EH with *kdoT* homolog from *R. rickettsii* strain 'Sheila Smith'. Synthesized by GenScript	This study, GenScript
pMMB::*kdoT*$_{D8A}$-His$_8$	pMMB::*kdoT*-His$_8$ with D8A mutation	This study
pMMB::*kdoT*$_{H13A}$-His$_8$	pMMB::*kdoT*-His$_8$ with H13A mutation	This study

pMMB::*kdoT*$_{H109A}$-His$_8$	pMMB::*kdoT*-His$_8$ with H109A mutation	This study
pMMB::*kdoT*$_{D110A}$-His$_8$	pMMB::*kdoT*-His$_8$ with D110A mutation	This study
pMMB::*kdoT*$_{R179A}$-His$_8$	pMMB::*kdoT*-His$_8$ with R179A mutation	This study
pMMB::*kdoT*$_{R180A}$-His$_8$	pMMB::*kdoT*-His$_8$ with R180A mutation	This study
pMMB::*kdoT*$_{R179K}$-His$_8$	pMMB::*kdoT*-His$_8$ with R179K mutation	This study
pMMB::*kdoT*$_{R180K}$-His$_8$	pMMB::*kdoT*-His$_8$ with R180K mutation	This study
pMMB::*kdoT*$_{R179Q}$-His$_8$	pMMB::*kdoT*-His$_8$ with R179Q mutation	This study
pMMB::*kdoT*$_{R180Q}$-His$_8$	pMMB::*kdoT*-His$_8$ with R180Q mutation	This study
pMMB::*kdoT*$_{R179/180A}$-His$_8$	pMMB::*kdoT*-His$_8$ with R179/180A mutation	This study
pMMB::*gnaT*$_{E283A}$-His$_8$	pMMB::*gnaT*-His$_8$ with E283A mutation	This study
pMMB::*gnaT*$_{E291A}$-His$_8$	pMMB::*gnaT*-His$_8$ with E291A mutation	This study
pMMB::*kdoT*/*gnaT*	pMMB67EH with *kdoT* and *gnaT*	(1)
pMMB::*kdoT*/*gnaT*$_{E283A}$	pMMB::*kdoT*/*gnaT* with GnaT E283A mutation	This study
**Primer**	**Sequence (5'→3')**	**Purpose**
pMMB-seq-F	TTGACAATTAATCATCGGCTCGTATAATGTGTGG	Plasmid cloning verification (pMMB)
pMMB-seq-R	ATTTGTCCTACTCAGGAGAGCGTTCAC	Plasmid cloning verification (pMMB)
EcoRI-kdoT-F-Ac	TAGCATGAATTCGGCTTATGGAGCTTTTatgCATATTGTCTATGTC	Cloning *kdoT* homolog from *A. calcoaceticus* into pMMB
BamHI-kdoT-R-Ac	TAGCATGGATCCAAATttaATTTTTCGCTAAAAGGTATGTTGCCACAC	Cloning *kdoT* homolog from *A. calcoaceticus* into pMMB
EcoRI-kdoT-F-An	TAGCATGAATTCTAGGGAGCTTTTatgCATATTGTCTATGTCTCTG	Cloning *kdoT* homolog from *A. nosocomialis* into pMMB
BamHI-kdoT-R-An	TAGCATGGATCCCGATGACAGCCAATGtcaTTTAGTGAGC	Cloning *kdoT* homolog from *A.*

		nosocomialis into pMMB
pET21-seq-F	GAGGATCGAGATCTCGATCCCGC	Plasmid cloning verification (pET21a)
pET21-seq-R	CAACTCAGCTTCCTTTCGGGCTTTG	Plasmid cloning verification (pET21a)
NdeI-kdoT-F	TAGCATCATATGCATATTGTCTATGTCTCTGATGGTAAAGCAGGG	Plasmid cloning (pET21a)
BamHI-kdoT-His$_8$-R	TAGCATGGATCCTTAGTGATGATGATGATGATGGTGGTGATTTTTCGCTAAAAGATATGTTGCCACACGG	Plasmid cloning (pET21a)
NdeI-kdoT-F	TAGCATCATATGAAAGTGATGCAACTTCTCCCAGAACTTAATAGC	Plasmid cloning (pET21a)
BamHI-gnaT-His$_8$-R	TAGCATGGATCCTTAGTGATGATGATGATGATGGTGGTGATTCAATACACTTTGATATAGCTCAAGGGTTTGGT	Plasmid cloning (pET21a)
kdoT-D8A-F	TTATGCATATTGTCTATGTCTCTGCGGGT	Site-directed mutagenesis
kdoT-D8A-R	GATGCCCTGCTTTACCCGCAGAG	Site-directed mutagenesis
kdoT-H13A-F	GTAAAGCAGGGGCGCGCTCACAAG	Site-directed mutagenesis
kdoT-H13A-R	GCCTAAAGCTTGTGAGCGCGCCC	Site-directed mutagenesis
kdoT-H109A-F	GCTGTGATTCCAGAGGCGGACG	Site-directed mutagenesis
kdoT-H109A-R	CAGGAATACCGTCCGCCTCTGGA	Site-directed mutagenesis
kdoT-D110A-F	GTGATTCCAGAGCACGCGGGTATTC	Site-directed mutagenesis
kdoT-D110A-R	CGCAGGAATACCCGCGTGCTC	Site-directed mutagenesis
kdoT-R179A-F	CATTTTAACGACTTCAGCGCGAACACC	Site-directed mutagenesis

kdoT-R179A-R	ACTCTGCTGGTGTTCGCGCTGA	Site-directed mutagenesis
kdoT-R179K-F	CATTTTAACGACTTCAAAGCGAACACC	Site-directed mutagenesis
kdoT-R179K-R	ACTCTGCTGGTGTTCGAAATGA	Site-directed mutagenesis
kdoT-R179Q-F	CATTTTAACGACTTCACAACGAACACC	Site-directed mutagenesis
kdoT-R179Q-R	ACTCTGCTGGTGTTCGAACTGA	Site-directed mutagenesis
kdoT-R180A-F	CATTTTAACGACTTCACGTGCGACACC	Site-directed mutagenesis
kdoT-R180A-R	ACTCCGCTGGTGTTCGACGTGAA	Site-directed mutagenesis
kdoT-R179A-F (double)	CATTTTAACGACTTCAGCGGCGACACC	Site-directed mutagenesis to generate R179/180A double mutant. Used with pMMB::*kdoT*-His_8 R180A template
kdoT-R179A-R (double)	ACTCTGCTGGTGTCGCCGCTGA	Site-directed mutagenesis to generate R179/180A double mutant. Used with pMMB::*kdoT*-His_8 R180A template
kdoT-R180K-F	CATTTTAACGACTTCACGTAAGACACC	Site-directed mutagenesis
kdoT-R180K-R	ACTCTGCTGGTGTAAAACGTGA	Site-directed mutagenesis
kdoT-R180Q-F	CATTTTAACGACTTCACGTCAGACACC	Site-directed mutagenesis
kdoT-R180Q-R	ACTCTGCTGGTGTAACACGTGA	Site-directed mutagenesis

gnaT-E283A-F	TCCAATCAAGCAGCAACATTCGGTAGAACA	Site-directed mutagenesis
gnaT-E283A-R	AATGCTGTTCTACCGAATGTTGCTGCTTG	Site-directed mutagenesis
gnaT-E291A-F	GGTAGAACAGCATTAGCAGCGCTCTCTG	Site-directed mutagenesis
gnaT-E291A-R	CCGACAGAGAGCGCTGCTAATGCT	Site-directed mutagenesis

References:

1. VanOtterloo LM, Macias LA, Powers MJ, Brodbelt JS, Trent MS. 2024. Characterization of Acinetobacter baumannii core oligosaccharide synthesis reveals novel aspects of lipooligosaccharide assembly. mBio 15:e03013-23.
2. Tucker AT, Nowicki EM, Boll JM, Knauf GA, Burdis NC, Trent MS, Davies BW. 2014. Defining Gene-Phenotype Relationships in Acinetobacter baumannii through One-Step Chromosomal Gene Inactivation. mBio 5:e01313-14.
